# Supplementary material for: Enhanced tumor targeting and timely viral release of mesenchymal stem cells/oncolytic virus complex due to GRP78 and inducible E1B55K expressions greatly increase the antitumor effect of systemic treatment
Source: Mol Ther Oncolytics. 2022 Sep 17;27:26–47. doi: 10.1016/j.omto.2022.09.004 (PMC9539630; doi:10.1016/j.omto.2022.09.004)
Supplement: Document S1. Figures S1, S2, Tables S1 and S2 [file mmc1.pdf]

OMTO, Volume *27*

Supplemental information

Enhanced tumor targeting and timely viral release of mesenchymal stem cells/oncolytic virus complex due to GRP78 and inducible E1B55K expressions greatly increase the antitumor effect of systemic treatment

Soojin Choi, Jeong A. Hong, Hye Jin Choi, and Jae J. Song

Table S1 List of PCR primers for subcloning

Vector Name	Forward sequence	Reverse sequence
pVAX1-3484-RFP	CCGGGTCGACGACATTGATT	GATTGTCGACCCATAGAGCC
pcDNA3.1-Hygro-E1B55K	GCGGATCCATGGAGCGAAGAAACCCATCT	GAGCGGCCGCTCAATCTGTATCTTCATCGCT
pFlag-CMV2-E4orf6	GTACAAGCTTATGACTACGTCCGGCGTTCC	CACCTCTAGACTACATGGGGGTAGAGTCAT
pCA14-E1B55K	TTCATCTAGAATGGAGCGAAGAAACCCATC	GACGAAGCTTTCAATCTGTATCTTCATCGC
pcDNA3.1-Hygro-GRP78	GATTCTCGAGATGAAGCTCTCCCTGG	GGCCTCTAGACTACAACTCATCTTTT
pRetro-Tetone-Puro-E1B55K	CCTCGTAAAGAATTCATGGAGCGAAGAAACC CATCTGAG	GAGGTGGTCTGGATCCTCAATCTGTATCTTCAT CGCTAGA

Table S2 List of qRT-PCR primers

Primer name	Forward sequence	Reverse sequence
Vimentin	TGGATTCACTCCCTCTGGTT	GGTCATCGTGATGCTGAGAA
β-catenin	TCTGAGGACAAGCCACAAGATTACA	TGGGCACCAATATCAAGTCCAA
HDAC6	AAGAAGACCTAATCGTGGGACT	GCTGTGAACCAACATCAGCTC
Daxx	GACGGACATTTCCTCTTCCA	TCTCATGCACTGACCTTTGC
Akt1	TCCTCCTCAAGAATGATGGCA	GTGCGTTCGATGACAGTGGT
MAPKAPK2	CAGCAGTTCCCGCAGTTC	CGAATTTCTCCTGGGTCCTC
PTEN	ACCAGAGACAAAAAGGGAGTA	ACCACAAACTGAGGATTGCA
TRAF6	CCTTTGGCAAATGTCATCTGTG	CTCTGCATCTTTTCATGGCAAC
STAT3	TATGCGGCCAGCAAAGAATCA	CGGGCAATCTCCATTGGCT
HSF1	GCCTTCCTGACCAAGCTGT	AAGTACTTGGGCAGCACCTC
HSP27	CTACATCTCCCGGTGCTTCA	GGATGGTGATCTCGTTGGAC
TGF-β1	CAAGGGCTACCATGCCAACT	AGGGCCAGGACCTTGCTG
GRP78	CTTGCCGTTCAAGGTGGTTG	GCATGGGTAACCTTCTTTCC
β-actin	ACTCTTCCAGCCTTCCTTC	ATCTCCTTCTGCATCCTGTC
E4orfB region	CGTGGTCAAACTCTACAGCC	GCATGAGCATGACTACGATG

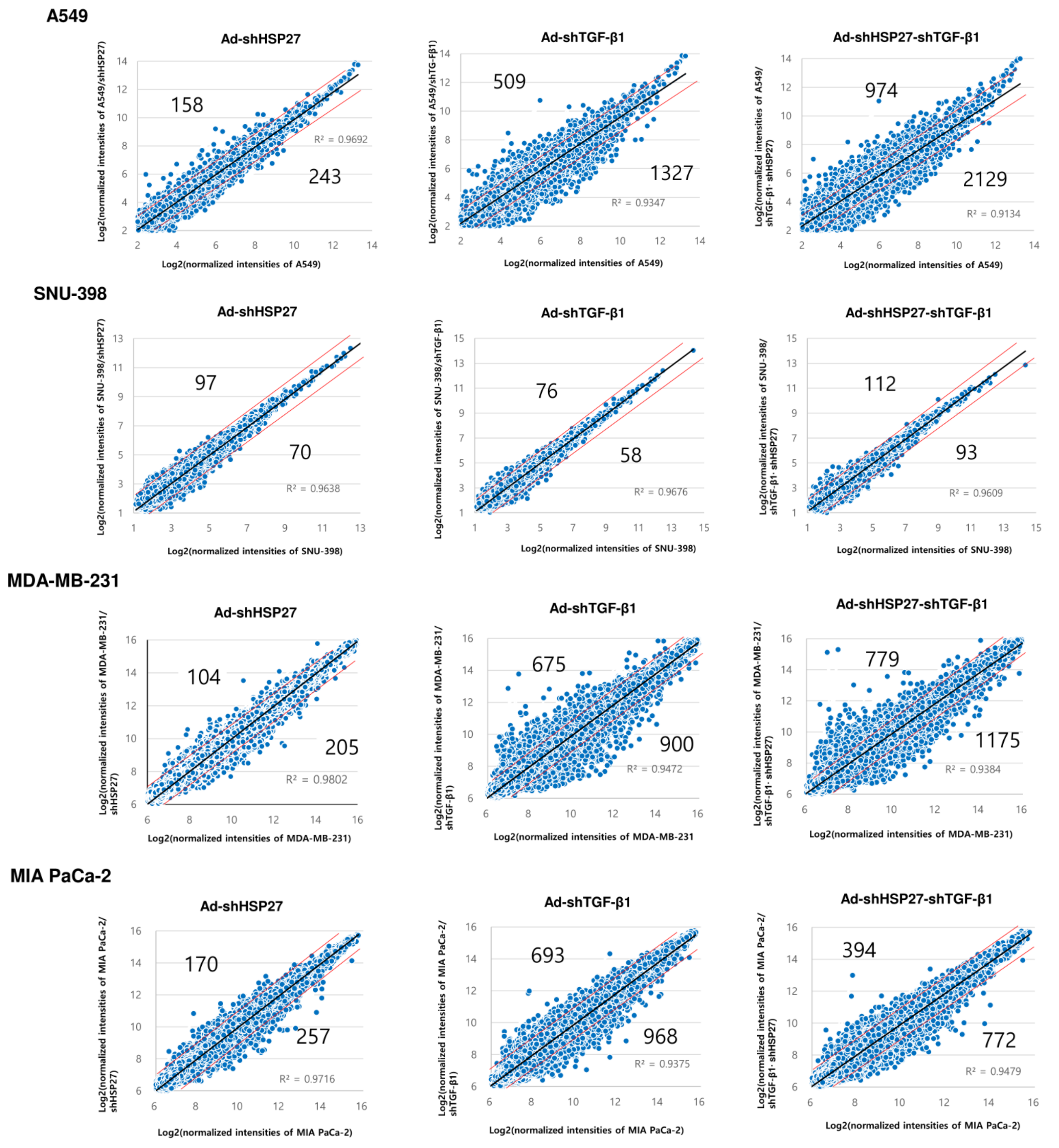

Figure S1 Scatter plots of relative probe set intensities after downregulation of HSP27, TGF-β1 or HSP27-TGF-β1 compared to before downregulation of HSP27, TGF-β1 or HSP27-TGF-β1, respectively, from four different cancer cells (A549, SNU-398, MDA-MB-231, MIA PaCa-2)
Normalized probe set intensities of before or after downregulation of HSP27, TGF-β1 or HSP27-TGF-β1 are plotted. The numbers indicate over 2-fold variation in intensities outside of two diagonal lines (red).

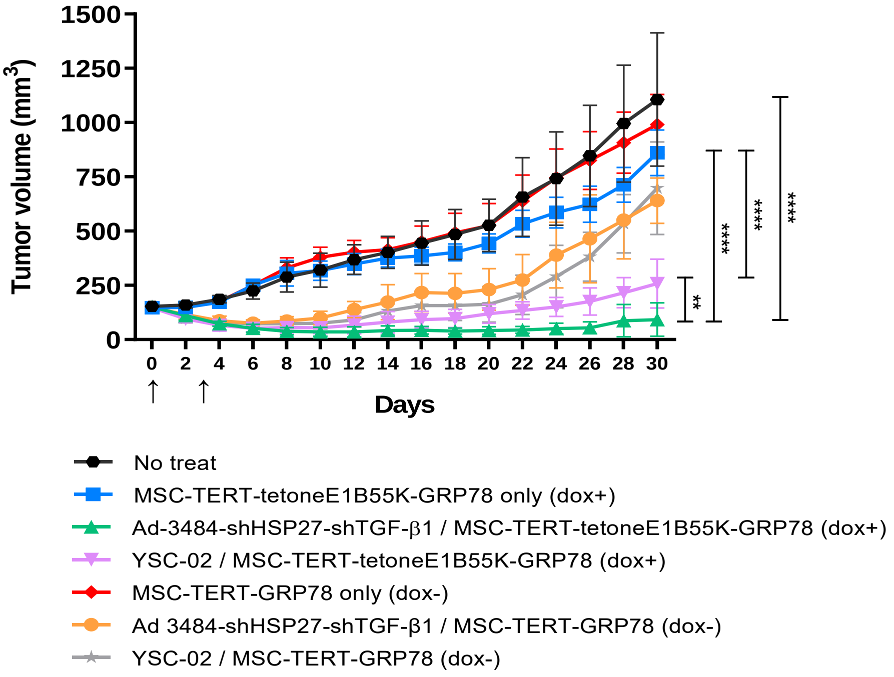

Figure S2 Tumor volume measurement in A549 xenograft models

8×10^6 A549 cells were implanted subcutaneously in the flank region of BALB/c athymic nude mice, and either MSC-TERT-GRP78 or MSC-TERT-tetoneE1B55K-GRP78 loaded with oncolytic adenovirus was injected intravenously twice with an interval of 3 days. Tumor volume was monitored and recorded every 2 days until the end of the study. Arrows indicate when the MSC or oncolytic adenovirus/MSC complex was administered. Error bars represent mean ± SD from 10 mice per group: **P<0.01, ****P<0.0001.
