# Supplementary material for: Enhanced tumor targeting and timely viral release of mesenchymal stem cells/oncolytic virus complex due to GRP78 and inducible E1B55K expressions greatly increase the antitumor effect of systemic treatment
Source: Mol Ther Oncolytics. 2022 Sep 17;27:26–47. doi: 10.1016/j.omto.2022.09.004 (PMC9539630; doi:10.1016/j.omto.2022.09.004)
Supplement: Document S2. Article plus supplemental information [file mmc2.pdf]

Molecular Therapy
Oncolytics
Original Article

Enhanced tumor targeting and timely viral release of mesenchymal stem cells/oncolytic virus complex due to GRP78 and inducible E1B55K expressions greatly increase the antitumor effect of systemic treatment

Soojin Choi,[1,2,5] Jeong A. Hong,[1,2,5] Hye Jin Choi,[3] and Jae J. Song[1,2,4,6]

[1]Severance Biomedical Science Institute, Yonsei University College of Medicine, Seoul 03722, Korea; [2]Institute for Cancer Research, Yonsei University College of Medicine, Seoul 03722, Korea; [3]Department of Internal Medicine, Yonsei University College of Medicine, Seoul 03722, Korea; [4]Graduate School of Medical Science, Yonsei University, Seoul 03722, Korea; [5]Graduate School of Medical Science, Brain Korea 21 Project, Yonsei University College of Medicine, Seoul 03722, Korea; [6]Dates Bio Co., Ltd., Seoul 03722, Korea

Systemic delivery of oncolytic viruses has been widely regarded as an impractical option for antitumor treatment. Here, we selected two target genes as leading components, and significant therapeutic effects were obtained by simultaneously reducing the expression of transforming growth factor β 1 (TGF-β1) and heat shock protein 27 (HSP27) in various cancer cell types. Downregulation of HSP27 reduced the cellular levels of tumor progression-related proteins, and the simultaneous downregulation of HSP27 and TGF-β1 increased tumor cell death beyond that observed with TGF-β1 downregulation alone. To increase the potential for systemic administration, we generated modified mesenchymal stem cells (MSCs) to act as oncolytic adenovirus factories and carriers and assessed bioavailability in tumors after MSC injection. The MSCs were modified to express 78-kDa glucose-regulated protein (GRP78) and adenovirus early-region 1B 55 kDa (E1B55K). The tightly controlled inducible system permitted selective timing of viral release from carrier MSCs within the tumor. This approach significantly improved viral production, tumor targeting, timely viral release at the tumor site, and antitumor efficacy of the oncolytic adenovirus. These combined results demonstrate that engineered MSCs can significantly enhance the antitumor effects of oncolytic viruses without adverse safety issues, which may greatly extend the clinical applicability of oncolytic adenoviruses.

INTRODUCTION

Oncolytic viruses are therapeutically useful because they selectively infect and damage cancerous tissues without harming normal tissues. Oncolytic viruses kill infected cancer cells in different ways, ranging from direct virus-mediated cytotoxicity to cytotoxic immune effector mechanisms.[1] Topical administration of oncolytic viruses has shown promising results in melanoma patients, but attempts at the systemic administration of oncolytic viruses targeting other tumor types have produced only rare and transient responses.[2] This limited effectiveness may be the result of insufficient viral delivery to tumors due to virus sequestration in the liver or spleen, virus recognition and elimination by the immune system, or difficulty crossing the endothelial barrier.[3,4] Therefore, the clinical applicability of oncolytic viruses could be increased by overcoming these barriers to their bioavailability in tumors, especially in patients for whom intratumor direct injection is not feasible or who have metastatic disease.[2]

Adenovirus serotype 5 (Ad5) is the most commonly used platform for therapeutic recombinant adenoviruses. Adults have a relatively high seroprevalence of anti-Ad5 antibodies due to natural infection.[5,6] Such pre-existing immunity can present a barrier to adenovirus vector-mediated gene transduction. In animal models, pre-immunization against adenovirus significantly reduces adenovirus vector-mediated gene transduction, and most intravenously injected adenoviruses are sequestered in the liver 30 min after injection.[7] One study reported that less than 0.001% of intravenously administered virions reached tumors 24 h after injection,[8] and another study reported 0.01% of injected oncolytic vesicular stomatitis virus in tumor tissues after 24 h,[9] suggesting that most injected viruses are rapidly cleared by the liver or immune system components, such as neutralizing antibodies.

Several approaches have exploited cell carriers to overcome these limitations of systemic oncolytic viral delivery. Mesenchymal stem cells

Received 12 May 2022; accepted 14 September 2022;
https://doi.org/10.1016/j.omto.2022.09.004.

Correspondence: Hye Jin Choi, Department of Internal Medicine, Yonsei University, 50-1 Yonsei-ro, Seodaemun-gu, Seoul 03722, Republic of Korea.
E-mail: choihj@yuhs.ac
Correspondence: Jae J. Song, Severance Biomedical Science Institute, Yonsei University, 50-1 Yonsei-ro, Seodaemun-gu, Seoul 03722, Republic of Korea.
E-mail: jjs109@yuhs.ac

This is an open access article under the CC BY license (http://creativecommons.org/licenses/by/4.0/).

(MSCs) are attractive candidates for delivery to tumor sites due to low immunogenicity and a tendency to migrate toward tumors; in addition, MSCs are technically simple to obtain and are associated with minimal ethical complications.[10,11] MSC-mediated oncolytic virus delivery may shield the virus from host defenses in transit.[8,10,12] However, few studies have investigated the therapeutic use of MSCs for systemic oncolytic virus administration, which may be due to poor tumor homing, inadequate oncolytic virus production or release, and the possible risk of MSC oncogenic potential after extended passage in culture.

Both intravenously administered and endogenous MSCs are recruited to the bloodstream, undergo a defined multistep process to exit circulation, and migrate to tumor sites. The homing of MSCs to tumor cells involves five steps: tethering and rolling, activation, arrest, transmigration or diapedesis, and migration.[13,14] During transmigration, MSCs travel through the endothelial cell layer and basement membrane, which is accomplished by the secretion of matrix metalloproteinases (MMPs) that break down the endothelial basement membrane.[14] One possible approach to overcoming existing clinical challenges is the use of engineered MSCs.

We considered enhancing the performance of MSCs by increasing the expression of 78-kDa glucose-regulated protein (GRP78, also called BiP or HSPA5), which is a stress-inducible chaperone in the endoplasmic reticulum (ER) that performs critical functions in maintaining ER homeostasis, assisting in protein folding and assembly, and exporting misfolded proteins for degradation.[15] GRPs can localize to the cell surface and regulate functions outside the ER that are important for health and disease. For example, GRP78 expression is directly correlated with tumor metastasis and promotes hepatocellular carcinoma cell invasion by increasing cell motility.[15] Similarly, induced GRP78 expression promotes colon cancer cell migration and invasion by upregulating MMP2, MMP9, and urokinase plasminogen activator (uPA).[16] We hypothesized that GRP78 expression could materially improve MSC homing to tumors.

We also considered enhancing MSC performance through the expression of adenovirus early-region 1B 55-kDa (E1B55K), which is a multifunctional phosphoprotein that promotes efficient viral replication.[17] During the early phase of Ad5 infection, E1B55K counteracts antiproliferative processes induced by the host cell, including the activation of both p53-dependent and p53-independent apoptosis, induction of cell-cycle arrest, and stimulation of cellular DNA damage responses. During the late phase of Ad5 infection, E1B55K controls late viral protein production by stimulating the cytoplasmic accumulation and translation of late viral mRNAs while simultaneously blocking host cell mRNA nuclear export and host protein synthesis.[18] These multiple functions of E1B55K require interaction with early-region 4 open reading frame 6 (E4orf6), which was recently shown to connect E1B55K to the cellular proteins cullin-5, Rbx1/RCO1/Hrt1, and Elongins B and C for the assembly of a Skp, cullin, F box (SCF)-like E3-ubiquitin-ligase complex that promotes proteasomal degradation of interacting factors p53, Mre11, DNA ligase IV, and integrin $\alpha3$ subunit.[19] We hypothesized that E1B55K expression could materially improve the MSC release of oncolytic virus at the tumor site.

In addition to improving viral vector delivery, the targeting of appropriate tumor genes is necessary to achieve the desired antitumor activity. Transforming growth factor β (TGF-β) is one of many targetable candidates and has pleiotropic functions regulating cell growth, differentiation, apoptosis, motility and invasion, extracellular matrix production, angiogenesis, and immune response.[20,21] TGF-β signaling pathway is complex and mediates both protumoral and antitumoral activities in cancer cells depending on their spatiotemporal context and microenvironment.[22] For example, in the tumor microenvironment, TGF-β suppresses immunosurveillance by inhibiting the infiltrating immune cells that mediate tumoricidal activities.[23,24] TGF-β has biphasic activity during tumorigenesis, suppressing tumorigenesis during the early stages but promoting tumor progression during the late stages. Consequently, many cancer therapies aim to downregulate TGF-β ligand expression.[25] However, recent studies in mouse models have shown that TGF-β inhibitors can unexpectedly induce metastasis in certain tumor types.[25,26] These observations suggest that the selection of targets with functions that complement TGF-β could also serve as a more effective antitumor strategy than targeting TGF-β, regardless of the tumor type or stage.[27] Therefore, we also considered heat shock protein 27 (HSP27) as an additional target gene in this study. HSP27 is a key regulator of protein folding and degradation in human cells.[28,29] Higher HSP27 expression has been observed in several human cancers with aggressive tumor behavior and poor prognosis, suggesting that reducing its expression could represent a promising therapeutic strategy.[29,30] Our recent data indicate that TGF-β1 downregulation induces HSP27 phosphorylation and reactive oxygen species (ROS) production, which leads to cancer cell death.[31] HSP27 is also involved in the epithelial-mesenchymal transition (EMT), metastasis, and invasion, processes that are also deeply related to TGF-β signaling.[29,32]

Together, these observations suggest that the simultaneous downregulation of both HSP27 and TGF-β1 could cooperatively enhance antitumor activity. To that end, we examined the effectiveness of using modified MSCs for the systemic delivery of an oncolytic adenovirus expressing small hairpin RNAs (shRNAs) targeting both HSP27 and TGF-β1 to determine whether this approach could improve the tumor bioavailability of systemically administered oncolytic adenovirus.

RESULTS

HSP27 and TGF-β1 downregulation reduces cancer progression-related proteins and promotes tumor cell death

Although HSP27 overexpression is commonly observed in various cancer cell types compared with normal cells, abundant HSP27 expression was also observed in normal cell lines (Figure 1A), suggesting that HSP27 may act as a survival-related protein rather than exclusively as a tumor progression marker. To evaluate the global transcriptomic impacts of reduced HSP27 and TGF-β1 expression,

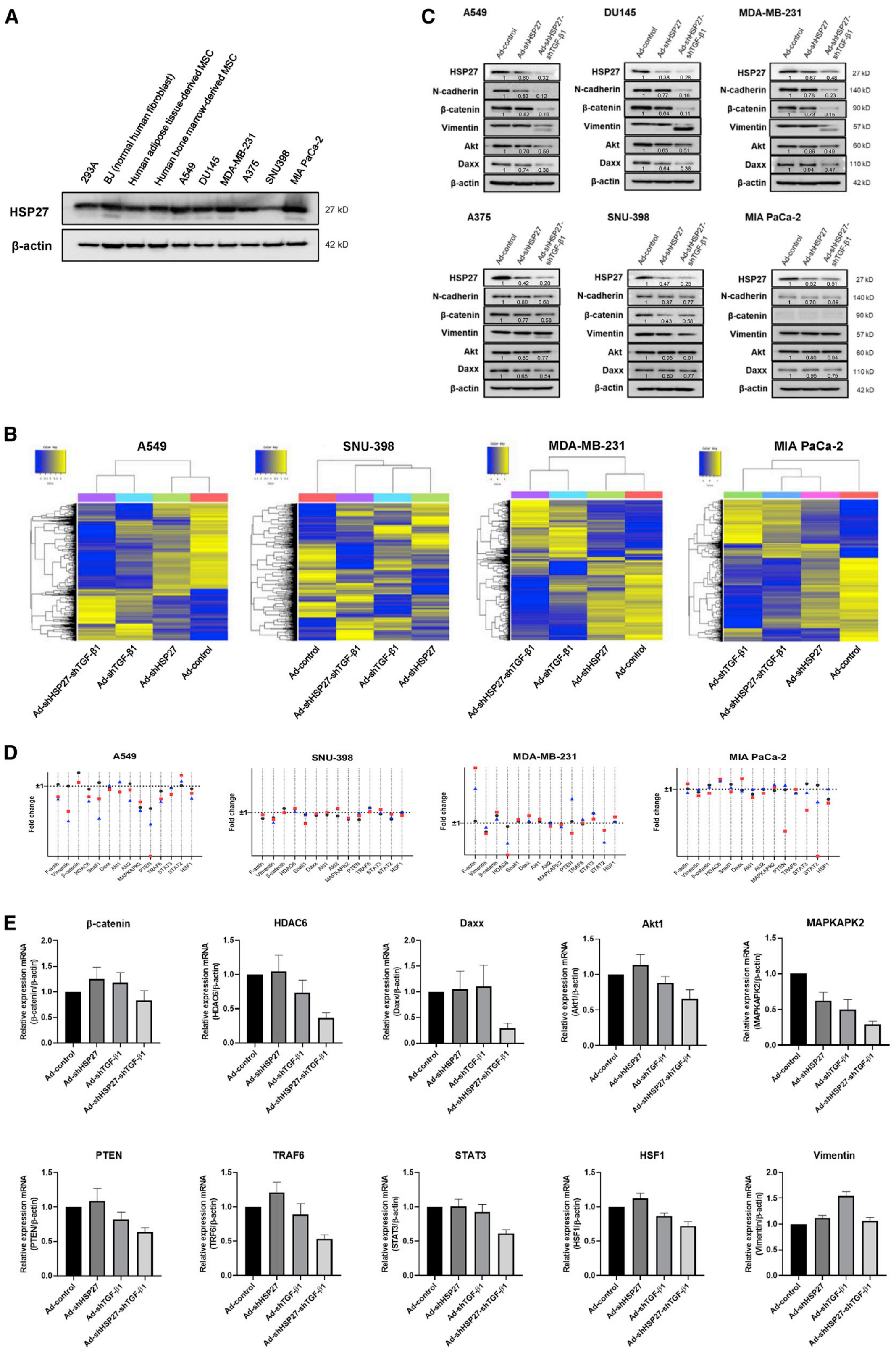

(legend on next page)

various cancer cell lines, including A549, SNU-398, MDA-MB-231, and MIAPaCa-2, were treated with shRNAs targeting TGF-β1 (shTGF-β1) and HSP27 (shHSP27), either alone or together (shHSP27-shTGF-β1), and assessed by microarray analyses using 47,316 probe sets. The results showed more than 1,500 (3.2%) differentially expressed genes with a greater than 2-fold change in expression in shTGF-β1-treated cells compared with untreated cells (Figure S1). We examined whether the number of genes with a greater than 2-fold change in expression following HSP27 downregulation was similar to the number identified for TGF-β1 downregulation, as previously shown.[33] Fewer genes were affected by HSP27 downregulation than were affected by TGF-β1 downregulation,[33] in all cell lines except SNU-398 cells (the most aggressive tumor cell line among the four examined), which showed very small numbers of genes with greater than 2-fold changes following TGF-β1 downregulation compared with normal cells (Figures 1B and S1). Although HSP27 downregulation resulted in different levels of gene fluctuation than TGF-β1 downregulation (less fluctuation in A549, MDA-MB-231, and MIA PaCa-2 cells and more fluctuation in SNU-398 cells), the levels of several putative HSP27 target proteins[32] were reduced by HSP27 downregulation, without significant reductions in the corresponding mRNA levels (Figures 1C and 1D), indicating that reduced protein levels as assessed by western blots were due to changes in HSP27 target protein translation or degradation.[32,34] We observed that the levels of several HSP27 chaperone proteins were further decreased by the simultaneous downregulation of both HSP27 and TGF-β1, including N-cadherin (Figure 1C), suggesting that putative HSP27 target proteins were also regulated by TGF-β1 at the transcriptional level (Figure 1D). Changes in the transcription levels of several putative HSP27 target proteins following TGF-β1 downregulation were individually confirmed using quantitative reverse transcriptase polymerase chain reaction (qRT-PCR), and the results showed similar patterns to those observed by microarray analysis, except for vimentin (Figure 1E), which may be regulated at the post-translational level.

Our prior studies targeted either TGF-β1 or HSP27 independently,[33,35,36] not both simultaneously. We previously showed that TGF-β1 downregulation substantially induces ROS generation and stress-activated protein kinase (SAPK) phosphorylation, accompanied by HSP27 phosphorylation, which leads to cell death.[33] Downregulation of both HSP27 and TGF-β1 synergistically decreased the expression of several tumor-related proteins, including Daxx, which is overexpressed in diverse cancers and is responsible for the repression of adenoviral replication (Figure 1C).[37–40] These synergistic activities were confirmed in clonogenic assays using various cancer cell types (Figure 2A), but differential clonogenic effects were observed across cancer cell types. These combined results indicate that shHSP27 and shTGF-β1 cooperatively reduce EMT-related hallmarks and survival signals, leading to cell death, regardless of the degree of cancer cell progression or aggressiveness.

Our results suggest that the tested shRNAs (shHSP27 and shTGF-β1) were essential for triggering the observed cancer cell death in our experiments. Therefore, we examined whether the simultaneous delivery of both shRNAs by an oncolytic adenovirus could efficiently reduce tumor growth *in vivo*. A schematic showing the structure of the oncolytic shHSP27-shTGF-β1 adenovirus construct and the effective repression of HSP27 and TGF-β1 transcripts by the oncolytic shHSP27-shTGF-β1 adenovirus are presented in Figures 2B and 2C, respectively. We confirmed that the simultaneous downregulation of TGF-β1 and HSP27 reduced Daxx levels beyond the reduction observed for either shRNA alone (Figure 1C). Increased viral replication due to the downregulation of both TGF-β1 and HSP27 increased cytotoxicity (Figure 2D). In two mouse models featuring tumors that developed from implanted cancer cells (MDA-MB-231 and MIA PaCa-2), the direct intratumoral injection of the shHSP27-shTGF-β1 adenovirus resulted in a strong antitumor effect; however, no tumor regression was observed in mouse models implanted with the aggressive SNU-398 cell line or the less aggressive A549 cell line following the intratumor injection of the shHSP27-shTGF-β1 adenovirus (Figure 2E). These findings indicate that the oncolytic shHSP27-shTGF-β1 adenovirus was able to confer an additional therapeutic effect associated with increased viral replication in an *in vivo* model.

MSCs derived from immortalized human adipose tissue have no passage limits and high adenovirus infectivity

The *in vivo* mouse model exhibited reduced tumor growth but not tumor regression following the intratumoral injection of the oncolytic shHSP27-shTGF-β1 adenovirus (Figure 2E). The viral administration route may have affected this outcome, as direct intratumoral injections can lead to suboptimal dosing due to backflow into the needle and insufficient viral distribution throughout the tumor.[41] By contrast,

Figure 1. HSP27 and TGF-β1 downregulation reduces cancer progression-related proteins and promotes tumor cell death

(A) Expression levels of HSP27 in diverse cell lines, including normal cells and cancer cells. (B) DEGs are shown in heatmaps. Gene expression levels are visualized as row-standardized *z* scores ranging from blue (−1) to yellow (+1) across all samples. Rows are organized by hierarchical cluster analysis with complete linkage and Euclidean distance as a measure of similarity from samples of A549, SNU-398, MDA-MB-231, and MIA PaCa-2. Each cell line was infected with adenovirus expressing HSP27 (Ad-shHSP27), TGF-β1 (Ad-shTGF-β1), or HSP27-TGF-β1 (Ad-shHSP27-shTGF-β1) shRNA at an MOI of 100. Adenovirus expressing nonsense shRNA (Ad-control) was used as the negative control, and each cell line was infected with Ad-control at an MOI of 100. The microarray data can also be found in the NCBI GEO database (GSE196290; GSE196290 is composed of GSE196286, GSE196287, GSE196288, and GSE196289). (C) Western blot analysis of cancer progression-related protein levels when each adenovirus was infected in various cancer cell types. The numbers represent relative western blot band intensities for Ad-control quantified using ImageJ software. (D) Gene fluctuation of HSP27 client proteins in four different cancer cell types after HSP27 downregulation (●), TGF-β1 downregulation (■), or HSP27-TGF-β1 downregulation (▲). Fold change (FC) was calculated by 2^(log2FC) to be converted to linear scale. Log2FC is the sum of normalized value of each downregulation minus normalized value of control. If the value is between 0 and 1, FC is calculated by a minus reciprocal −1/FC). Actually, FC 1 or −1 means no change in gene fluctuation. (E) Transcriptional regulation of putative HSP27 client proteins by TGF-β1 downregulation was individually confirmed using qRT-PCR. mRNA levels of various target proteins were examined by using various qRT-PCR primers (Table S2) after infection of adenovirus expressing shRNAs targeting HSP27, TGF-β1, or both HSP27 and TGF-β1 (100 MOI).

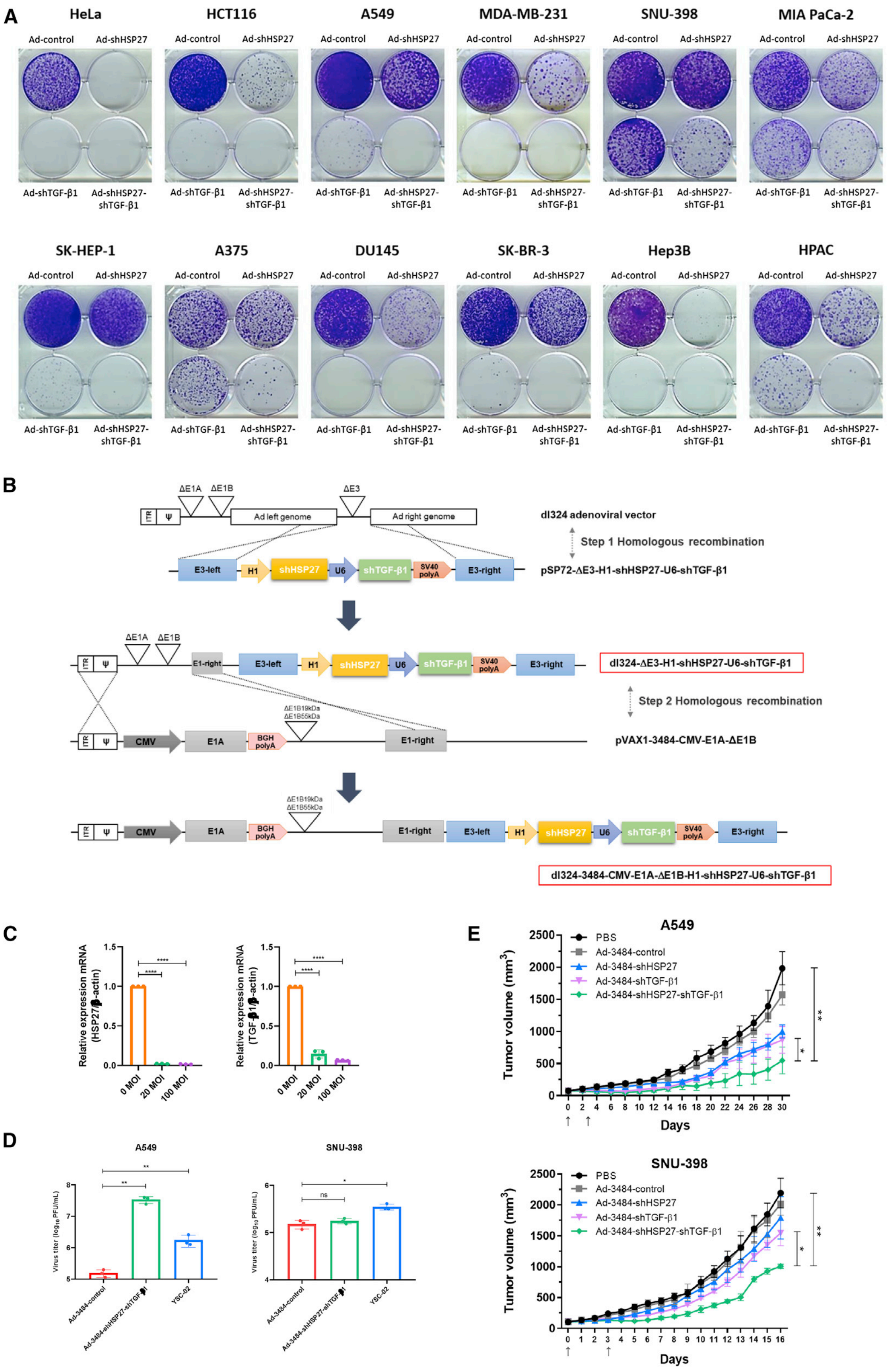

(legend on next page)

systemic intravascular viral delivery can achieve widespread viral distribution throughout the tumor.[41] However, the systemic delivery of a naked virus can induce peripheral organ toxicity, immune-mediated virus inactivation, or nonspecific uptake by other tissues, such as the liver and spleen.[41,42] To overcome these challenges, MSCs were used as virus carriers and amplifiers with tumor-homing properties, acting to shield the virus from the immune system while traversing through the bloodstream.[8,41] MSCs were engineered to deliver the telomerase reverse transcriptase (TERT) gene via retroviral infection, and selected clones expressing TERT facilitated passage extension during large-scale MSC production. We confirmed that MMP1 and GRP78 expression levels, which are important factors for MSC homing efficiency, were not reduced in TERT-expressing MSC clone 22 (Figure 3A). The viral infectivity of MSC-TERTs was examined using a red fluorescent protein (RFP)-expressing, replication-incompetent adenovirus. This strategy allowed for extended passages, from 10 to at least 30, and increased infectivity from 30%–40% to 80%–90% (Figure 3B).

E1B55K expression contributes to enhanced adenoviral replication in MSCs

We previously reported that exogenous E1B55K expression caused a dramatic increase in adenoviral replication (up to 100-fold) in mouse cells.[43] To further investigate whether MSCs could function as an adenoviral amplification reservoir after the introduction of E1B55K, we examined whether the E1B55K expression level in MSC-TERTs increased in a dose-dependent manner according to the multiplicity of infection (MOI) (Figure 3C). In this study, virus production in MSC-TERTs increased by more than 100-fold after infection with an E1B55K-expressing adenovirus compared with control MSC-TERTs without E1B55K expression (Figure 3D). A similar dose-dependent response was observed following infection with the oncolytic adenovirus expressing shHSP27-shTGF-β1 (Figure 3D). The viral production of oncolytic adenoviruses expressing either RFP or shHSP27-shTGF-β1 increased over time in cells expressing E1B55K compared with cells without E1B55K expression (Figure 3E).

E1B55K expression alone augments p53 accumulation rather than p53 degradation

We failed to generate a stable MSC line expressing E1B55K using a retroviral system. Therefore, we investigated whether E1B55K had other relevant biological functions and found that MSCs expressing E1B55K demonstrated p53 accumulation and reduced levels of corresponding survival signals (Figure 4A). We introduced a doxycycline-inducible gene expression system called pRetroX-TetOne-Puro to express E1B55K. The TetOne 3G transactivator protein is expressed constitutively by the human phosphoglycerate kinase (PGK) promoter but is unable to bind to the TRE3G promoter in the absence of doxycycline. Once bound to doxycycline, the transactivator undergoes a conformational change, binds to the TRE3G promoter, and activates transcription of E1B55K (Figure 4B). We confirmed that E1B55K expression was only induced in the presence of doxycycline (Figure 4C, lane 5), indicating no leak-through expression. Additionally, it was inducible at low doxycycline concentrations (Figure 4D, lane 4). The previously observed increase in viral production associated with E1B55K expression (Figure 3D) was also observed with the pRetroX-TetOne vector system in the presence of doxycycline (Figure 4E). After confirming control of the E1B55K-inducible regulatory system, we generated an inducible gene expression construct to stably express E1B55K in MSCs (MSC-TERT-tetoneE1B55K; Figures 4F and 4G). MSC-TERT-tetoneE1B55K clones consistently exhibited p53 accumulation under the inducible expression of E1B55K (Figure 4G). The observation of E1B55K-induced p53 accumulation differed from previous studies,[44,45] which reported that E1B55K expression induced p53 degradation via the formation of E4orf6-E1B55K complexes functioning as E3 ubiquitin ligases. Therefore, we investigated whether introducing E4orf6 expression into E1B55K-expressing MSCs reduced p53 accumulation. The results showed that exogenous E4orf6 expression reduced E1B55K-induced p53 accumulation in a dose-dependent manner (Figure 4H), suggesting that E4orf6 functions as an E1B55K inhibitor. We observed that E4orf6 inhibited E1B55K-induced p53 accumulation at the transcriptional level using a p53-specific promoter inserted into pGL2 (pGL2-356bp, Addgene #16292, p53 promoter fragment; Figure 4I). E4orf6 may inhibit the binding of E1B55K with the p53 promoter, leading to decreased p53 expression and the repression of viral production in the presence of doxycycline (Figure 4J). After virus replication and assembly are completed, viral progeny cannot be released until cells undergo apoptosis; therefore, the inhibition of apoptosis may also reduce viral production. In addition, the E4orf6-E1B55K complex was previously shown to induce the proteasomal degradation of p53. Taken together, these results and our previous work support the possibility that the relationship between E1B55K and p53 accumulation promotes viral production in MSCs.[46]

GRP78 facilitates tumor trafficking of MSCs

The main barriers to systemic delivery of oncolytic viruses to tumors are virus sequestration by other organs, such as the liver, spleen, and

Figure 2. Downregulation of HSP27 and TGF-β1 demonstrates antitumorigenic activities in various cancer cell types

(A) Cancer cell lines were treated with Ad-control, Ad-shHSP27, Ad-shTGF-β1, and Ad-shHSP27-shTGF-β1. After 48 h, cells were redistributed and incubated for an additional 7–14 days for clonogenic assays. When the cells formed several colonies, they were fixed and stained. (B) Structure of oncolytic adenoviral vector carrying two therapeutic target regions expressing shRNAs of HSP27 and TGF-β1. (C) HSP27 and TGF-β1 downregulation by Ad-3484-shHSP27-shTGF-β1 at different MOIs. A549 cells were infected with the indicated MOIs of oncolytic adenovirus expressing shRNAs targeting HSP27 and TGF-β1. Expression of HSP27 and TGF-β1 by qRT-PCR is shown. Error bars are mean ± SD from three independent experiments: ****$p < 0.0001$. (D) Quantification of infectious virus particles released from A549 (left) and SNU-398 (right). Each cell was infected with Ad-3484-control, Ad-3484-shHSP27-shTGF-β1, or YSC-02 at an MOI of 100 for 4 h. After 48 h, all virus particles released from the cells were collected and the amount of infectious virus particles were calculated. Error bars are mean ± SD from three independent experiments: *$p < 0.05$; **$p < 0.01$; ns, not significant. (E) Tumor volume measurements in A549 (upper) and SNU-398 (lower). Each tumor was transplanted subcutaneously in the flank region of BALB/c athymic nude mice, and PBS or each indicated virus was injected intratumorally twice with an interval of 3 days. Arrows indicate when the virus was administered. Tumor volume was monitored and recorded every 2 days until the end of the study. Error bars represent mean ± SD from five mice per group: *$p < 0.05$, **$p < 0.01$.

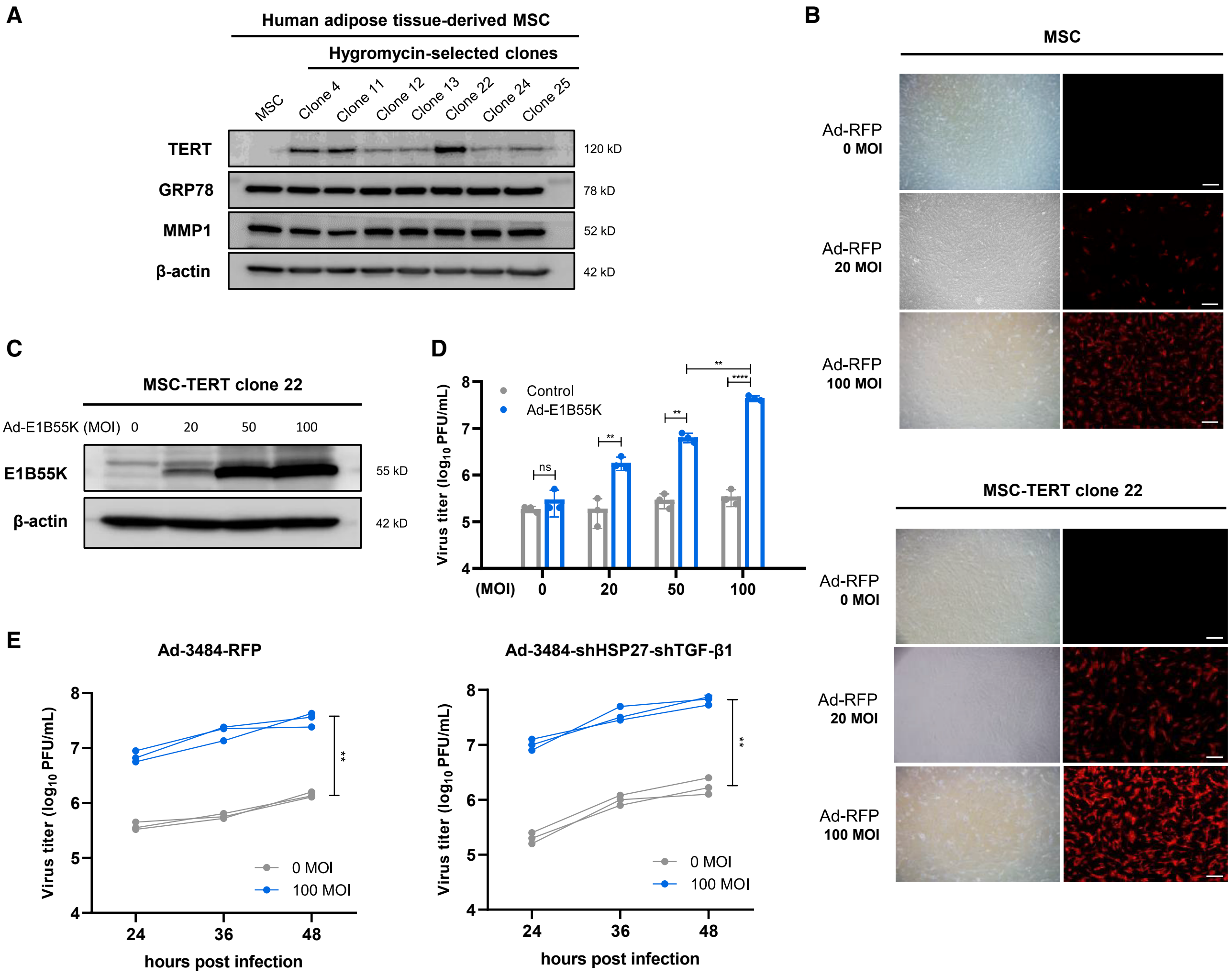

Figure 3. Immortalized human adipose tissue-derived MSCs have no passage limit and E1B55K in *trans* contributes to an enhanced adenoviral replication in MSCs

(A) TERT expression in hygromycin-selected MSC clones. Every selected clone was cultured in duplicate, and one of the duplicate cultures was lysed for western blot analysis. (B) MSC and MSC-TERT clone 22 were infected with RFP-expressing replication-incompetent adenovirus at the indicated MOIs. After 48 h, RFP expression was observed with fluorescence microscope excitation at 540–585 nm and emission at 600 nm. Scale bars, 50 μm. (C) MSC-TERT was infected with Ad-E1B55K at the indicated MOIs. After 48 h, E1B55K expression was analyzed by western blot. (D) Virus production is related to E1B55K expression levels in MSCs. MSC-TERT was sequentially infected with the indicated MOIs of Ad-control or Ad-E1B55K and 100 MOI of Ad-3484-shHSP27-shTGF-β1 for 4 h. After 48 h, all virus particles released from MSC-TERT were collected and the amount of infectious virus particles was quantified. Error bars are mean ± SD from three independent experiments: $**p < 0.01$; $****p < 0.0001$; ns, not significant. (E) Time-dependent virus production with or without E1B55K expression in MSCs. MSC-TERT was sequentially infected with 0 or 100 MOIs of Ad-E1B55K and 100 MOIs of Ad-3484-RFP (left) or Ad-3484-shHSP27-shTGF-β1 (right) for 4 h. After 24, 36, and 48 h, all virus particles released from MSC-TERT were collected and the amount of infectious virus particles was quantified. Data from three independent experiments are shown. $**p < 0.01$.

lung; virus neutralization by pre-existing antibodies; and complement activation in the bloodstream.[7,42] We contemplated how to overcome these hurdles and chose to use MSCs as oncolytic virus carriers due to their tumor-homing abilities; however, only a small percentage of MSCs reach the targeted tumor tissue after systemic administration.[13,14] Thus, we sought to identify factors that would allow MSCs to migrate more effectively along blood vessels to tumor sites and increase MSC transmigration into tumor tissues. We considered stromal cell-derived factor 1 (SDF-1) and insulin-like growth factor 1 (IGF-1), which are involved in MSC homing.[47–50] These factors are both known to promote MSC proliferation and migration through the hypoxia-inducible factor 1 alpha (HIF-1α)-GRP78-Akt signaling axis.[51,52] Based on previous studies demonstrating that GRP78 is associated with tumor metastasis and invasion through increased cell motility, we investigated whether GRP78 could enhance the tumor-homing abilities of MSCs.[53–55] SDF-1 preconditioning did not increase C-X-C motif receptor 4 (CXCR4) expression in MSCs, and pretreatment of MSCs with IGF-1 resulted in no significant change

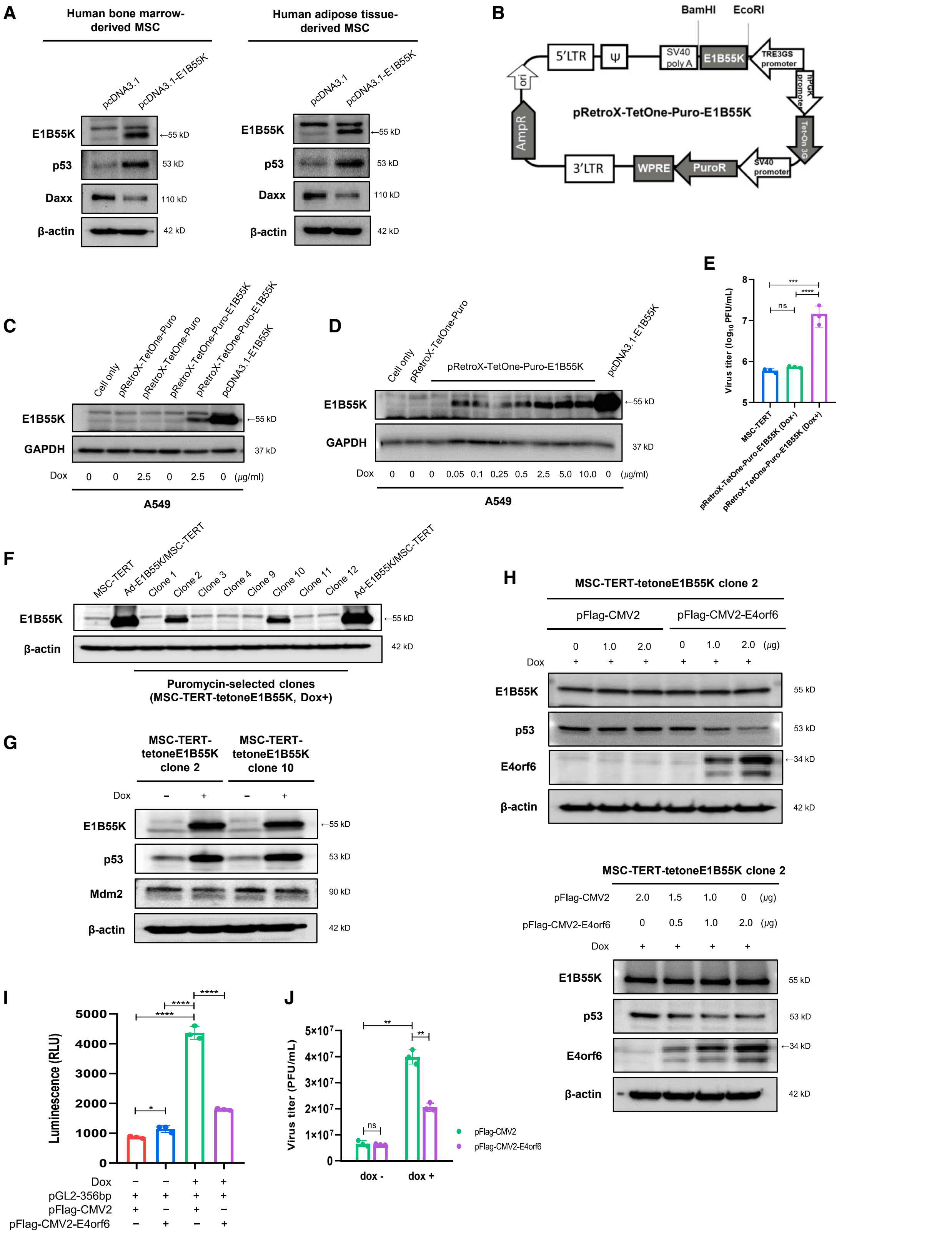

(legend on next page)

in the expression levels of homing-related proteins (data not shown). However, GRP78-overexpressing MSCs displayed an overall increase in the expression of various homing markers, including CXCR4, CD44, MMP1, and MMP2 (Figure 5A).[16,56–61] MMP2 is a critical molecule involved in the transmigration step of the MSC homing mechanism that was upregulated in several MSC-TERT-GRP78 clones (Figure 5B). Therefore, we selected two clones stably overexpressing GRP78 and MMP2 for further experiments.

To confirm tumor-homing activity *in vivo*, SNU-398 cells were subcutaneously implanted into the shoulder region of mice to distinguish luciferase activity from those found in other internal organs, such as the liver or lung. After the development of tumors measuring 150 mm^3, MSC-TERT or MSC-TERT-GRP78 clones infected with luciferase-expressing adenovirus (Ad-Luc) were injected into mice through the tail vein. The results showed that the most robust luciferase activity was localized to the expected tumor region within 6 h after tail vein injection, particularly in mice injected with MSC-TERT-GRP78 clones. We verified that trafficking was specific to tumors, not to other organs (Figure 5C). Analysis of the biodistribution of MSC-TERT-GRP78 clones carrying the oncolytic adenovirus expressing shHSP27 and shTGF-β1 indicated that most of the adenovirus localized to tumor tissues and was rarely detected in other organs (10^{10} copies in tumor tissues versus 100 copies in other organs; Figure 5D). Variations in adenoviral distribution, even among tumor tissues, were likely due to inefficient viral production and oncolysis in the SNU-398 tumor model without the E1B55K regulatory system to ensure timely viral release. To explore the biodistribution of MSC-TERT-GRP78 clones expressing luciferase after tail vein injection, lung, liver, and subcutaneous tumor tissues were harvested separately at 6 and 24 h after tail vein injection, and luciferase activities were examined. Luciferase activities were primarily detected in tumor tissues and were rarely detected in the lung or liver at 6 or 24 h after tail vein injection, which was similar to the observed viral gene distribution shown in Figure 4D (Figure 5E). Additionally, tumor targeting was greatly increased by GRP78 expression compared with MSC-TERTs without GRP78 expression (50- to 100-fold increase in tumor localization; Figure 5E).

MSC-TERT-tetoneE1B55K-GRP78 significantly enhances the *in vivo* antitumor efficacy of oncolytic adenovirus

We generated MSC-TERT-tetoneE1B55K clones that overexpressed GRP78 (Figure 6A). A clone with stable GRP78 and MMP2 overexpression was selected, and doxycycline was found to efficiently induce E1B55K expression without leakage in this clone (Figure 6B). As expected, doxycycline also increased viral production by more than 10-fold in the selected MSC-TERT-tetoneE1B55K-GRP78 clone (final clone 21; Figure 6C). We further examined the *in vitro* viral production kinetics after infection of the MSC-TERT-tetoneE1B55K-GRP78 clone with the oncolytic adenovirus, followed by treatment with doxycycline. Determining the appropriate time point for doxycycline treatment is critical for maximizing viral release after MSCs home to the tumor. Therefore, the kinetics of viral production and the viability of MSCs infected with the oncolytic adenovirus were examined. We observed that viral production in MSCs was maximized after 48 h of infection with doxycycline treatment, at which time most MSCs were eliminated due to oncolysis (Figures 6D and 6E). The rapid localization of MSCs to the tumor site within 6 h greatly simplified the identification of the optimal doxycycline treatment time to achieve maximal oncolytic virus release from intravenously injected MSCs infected with oncolytic virus. We confirmed that rapid and accurate tumor trafficking occurred consistently in tumors derived from multiple implanted cell types, including SNU-398, A549, and MIA PaCa-2 cells (Figure 6F). Tumor infiltration by MSCs was assessed using cell tracker red-labeled MSCs, which showed that GRP78 overexpression enhanced MSC penetration into interior tumor tissues, whereas MSC-TERT clones typically remained on the tumor tissue periphery (Figure 6G). We next performed an *in vivo* antitumor efficacy test using the final clone

Figure 4. E1B55K alone increases p53 accumulation rather than p53 degradation

(A) Human bone marrow-derived MSCs (left) and human adipose tissue-derived MSCs (right) were transfected with 2 μg of pcDNA3.1-Hygro or pcDNA3.1-Hygro-E1B55K plasmid. After 48 h, protein expression was analyzed by western blot. (B) Schematic structure of pRetroX-TetOne-Puro-E1B55K. (C) A549 was transfected with 2 μg of pRetroX-TetOne-Puro, pRetroX-TetOne-Puro-E1B55K, or pcDNA3.1-E1B55K, and then cells were treated with or without 2.5 μg/mL doxycycline. After 48 h, cells were lysed for detection of E1B55K expression levels by western blot. (D) Induction of E1B55K expression in A549 in the presence of various concentrations of doxycycline. A549 was transfected with 2 μg of pRetroX-TetOne-Puro, pRetroX-TetOne-Puro-E1B55K, or pcDNA3.1-E1B55K, and then, only cells transfected with pRetroX-TetOne-Puro-E1B55K were treated with doxycycline at indicated concentrations. After 48 h, cells were lysed for detection of E1B55K expression levels by western blot. (E) Measuring virus production levels in MSC-TERT transfected with pRetro-X-TetOne-Puro-E1B55K. Cells were transfected with 1 μg of pRetroX-TetOne-Puro-E1B55K, and then infected with 100 MOIs of Ad-3484-RFP. After 4 h of infection, cells were treated with or without 0.5 μg/mL doxycycline. After 48 h, all virus particles released from the cells were collected and the amount of infectious virus particles was calculated. Error bars are mean ± SD from three independent experiments: ***$p < 0.001$; ****$p < 0.0001$; ns, not significant. (F) Screening for inducible expression of E1B55K in puromycin-resistant clones in the presence of 0.5 μg/mL doxycycline. Positive clones for inducible expression of E1B55K were identified using MSC-TERT as a negative control and MSC-TERT infected with Ad-E1B55K (Ad-E1B55K/MSC-TERT) as a positive control. Ad-E1B55K/MSC-TERT was loaded on both ends to more clearly confirm the expression of E1B55K. (G) Protein expression in MSC-TERT-tetoneE1B55K clones treated with or without doxycycline for 36–48 h. (H) MSC-TERT-tetoneE1B55K clone 2 was transfected with pFLAG-CMV2 or pFLAG-CMV2-E4orf6 plasmid at indicated concentrations. Then, doxycycline was added to induce E1B55K expression. After 48 h, cells were lysed and protein expression levels were detected by western blot. (I) Measuring p53 promoter activity in MSC-TERT-tetoneE1B55K clone 2 by luciferase reporter assay. Cells were cotransfected with pGL2-356bp and pFLAG-CMV2 or pGL2-356bp and pFLAG-CMV2-E4orf6 (1 μg of each plasmid). Then, cells were treated with or without doxycycline for 48 h to induce E1B55K expression. The transcriptional activity of p53 was shown based on the measured firefly luciferase activities. Error bars are mean ± SD from three independent experiments: *$p < 0.05$, ****$p < 0.0001$. (J) Measurement of virus production in MSC-TERT-tetoneE1B55K clone 2. Cells were transfected with 1 μg of pFLAG-CMV2 or pFLAG-CMV2-E4orf6 and then infected with 100 MOIs of Ad-3484-shHSP27-shTGF-β1. After 4 h of infection, cells were treated with or without doxycycline. After 48 h, all virus particles released from the cells were collected and the amount of infectious virus particles was calculated. Error bars are mean ± SD from three independent experiments: **$p < 0.01$; ns, not significant.

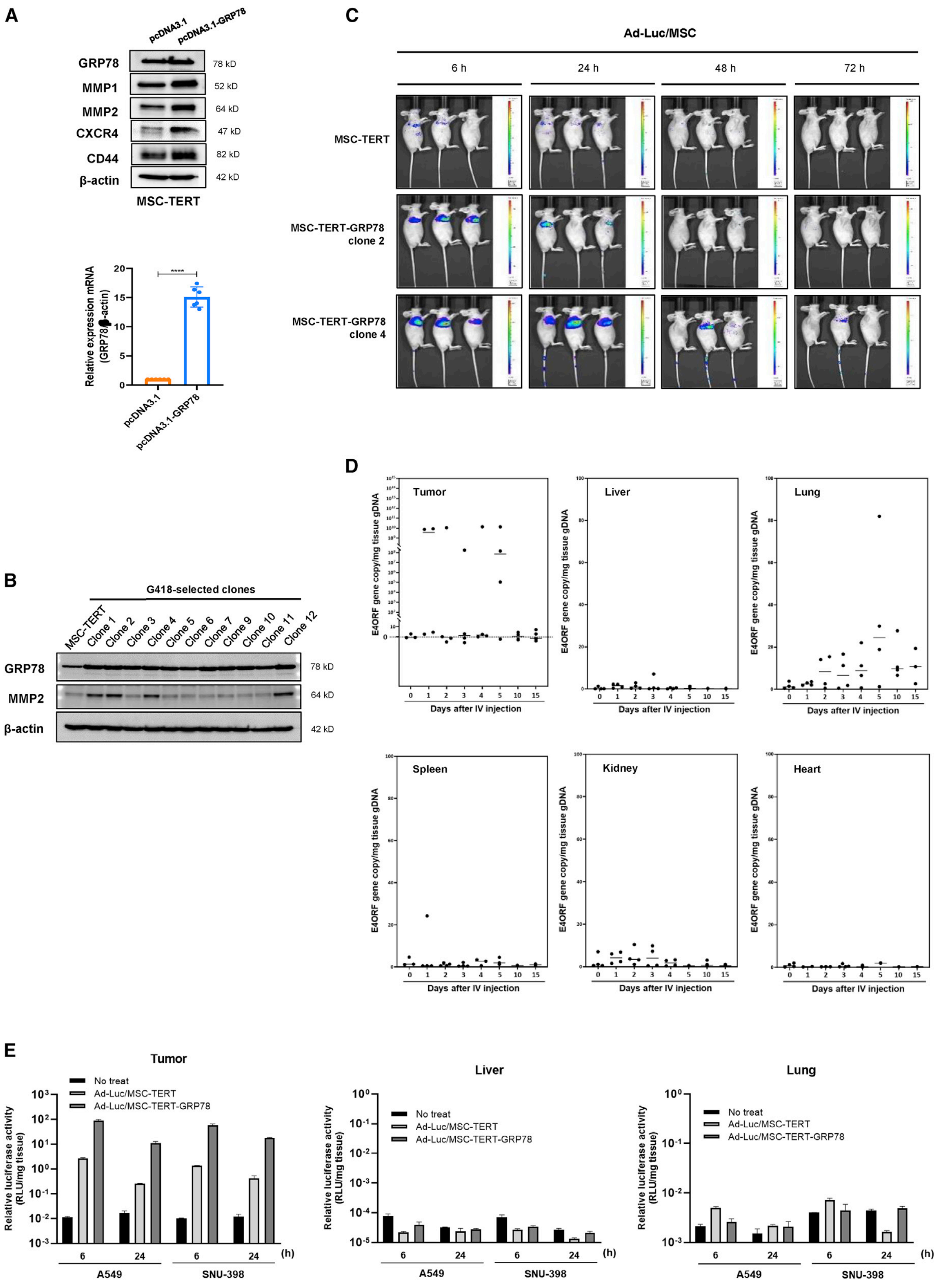

(legend on next page)

21 infected with two different oncolytic adenoviruses: one expressing shHSP27-shTGF-β1 and one expressing granulocyte-macrophage colony-stimulating factor (GM-CSF)-FMS-like tyrosine kinase 3 ligand (Flt3L)-tumor necrosis factor-related apoptosis-inducing ligand (TRAIL), in addition to shHSP27-shTGF-β1 (named YSC-02). The overall scheme of the animal study using MSC clones infected with oncolytic adenoviruses is presented in Figure 7A. The intravenous injection of MSC clones infected with either oncolytic adenovirus in A549 xenograft mouse models resulted in significant tumor regression compared with mice treated with oncolytic adenoviruses by direct intratumor injection (Figures 2E and 7B–7D). Mice treated with MSCs infected with YSC-02 exhibited a slight increase in tumor volume compared with mice treated with MSCs infected with the shHSP27-shTGF-β1 adenovirus, suggesting that virus types should be matched with specific cancer cells. Differential viral production appears to depend on the virus type (shHSP27-shTGF-β1 versus YSC-02) and the cancer cell type (A549 versus SNU-398) (Figure 2D). The inducible regulatory system controlling E1B55K expression allowed for increased viral production and release at the tumor site in the presence of doxycycline, and timely viral release was evident compared with oncolytic adenovirus-infected MSC-TERT-GRP78 clones lacking E1B55K expression (Figure S2, green triangle versus orange circle). Immunohistochemistry analysis after a second injection with MSCs carrying oncolytic adenoviruses indicated that adenovirus could be detected in every sample examined (Figure 7E), providing strong evidence that tumor regression occurred due to robust viral production and oncolysis. Potential clinical applicability was assessed by reducing the dose of MSC clones infected with the oncolytic adenovirus after tumor regression to assess antitumor efficacy at lower dosages. Significant tumor regression continued to be observed when using only one-fifth of the initial dose, and one mouse presented with complete remission (Figures 7F and 7G). Additionally, no significant abnormal activities, sudden weight loss, or acute deaths were observed among treated mice for 29 days after intravenous injection, even in the group receiving multiple high-dose administrations of MSCs infected with oncolytic adenoviruses (Figures 8A and 8B).

DISCUSSION

Systemic delivery of oncolytic viruses has become an important goal for treating human cancers resistant to current treatment modalities due to the antigen-agnostic cancer vaccine nature of these viruses.[62] Several studies seeking to overcome current limitations to the systemic delivery of oncolytic viruses have recently been reported.[63–65] Atasheva et al.[63] reported the targeted mutagenesis of functional sites in the adenovirus capsid to enhance resistance to inactivation by blood factors, avoid sequestration by the liver and macrophages, and reduce hepatotoxicity following intravenous delivery. Zhang et al.[64] reported the systemic administration of MSCs infected with oncolytic adenoviruses carrying IL-24/endostatin for improved glioma therapy. However, these reported approaches also have drawbacks, including limited viral biodistribution, viral sequestration in the livers of experimental mice (e.g., NOD SCID gamma (NSG) or non-obese diabetic/severe combined immunodeficiency (NOD/SCID)), and the presence of neutralizing antibodies.[63] Additional limitations that must be carefully addressed include tumor retardation without clear regression; safety issues associated with residual, uninfected MSCs; and the pulmonary first-pass effect, in which available MSC numbers are reduced due to MSC sequestration in the lung. In addition, treatment of glioma must overcome the challenge of crossing the blood-brain barrier.[64] The safety issue related to the first-pass effect in the lung was not mentioned either in Yoon et al.[65]

In this study, we examined modified MSCs infected with oncolytic adenoviruses as a candidate anticancer treatment. Our work contributes to circumventing important barriers to oncolytic virus effectiveness by improving tumor targeting and regulating the timing of viral release. These improvements have been achieved without additional toxicity issues and without major limitations, including the pulmonary first-pass effect, which are fundamental barriers that have arisen in previous clinical trials of stem cell delivery.[66,67] The small size of MSCs likely contributed to overcoming the pulmonary first-pass effect. The modified MSCs used in this study are less than 5 μm in diameter, which is smaller than the diameter of red blood cells (7.2–8.4 μm), allowing the MSCs to pass through lung capillaries more readily. Additionally, key molecules were identified, including GRP78 and E1B55K, the manipulation of which resulted in marked improvements in tumor targeting and the control of viral release from MSCs, respectively. GRP78 overexpression increased the rate and accuracy of tumor targeting by MSCs expressing oncolytic adenoviruses (50- to 100-fold) and produced a true Trojan horse effect

Figure 5. GRP78 facilitates tumor trafficking of MSCs

(A) MSC-TERT was transfected with 2 μg of pcDNA3.1-Hygro or pcDNA3.1-Hygro-GRP78 plasmid. After 48 h, protein expression was analyzed by western blot (upper), and GRP78 mRNA expression was measured by qRT-PCR (lower). Error bars are mean ± SD from three independent experiments: ****$p < 0.0001$. (B) Expression levels of GRP78 and MMP2 in G418-selected MSC-TERT-GRP78 clones. Clones stably overexpressing GRP78 and MMP2 were used for further experiments. (C) Bioluminescence imaging of MSC-TERT or MSC-TERT-GRP78 (clone 2 and clone 4) loaded with Ad-Luc in mice bearing SNU-398 tumors. MSC-TERT and MSC-TERT-GRP78 distribution was monitored *in vivo* using the IVIS Spectrum System at 6, 24, 48, and 72 h after tail vein injection of Ad-Luc-loaded MSCs. Each scale bar shows the intensity of bioluminescence; minimum and maximum radiances are indicated. (D) Quantification of adenovirus copy numbers in tissues by qRT-PCR analysis. BALB/c athymic nude mice were transplanted with SNU-398 cells by subcutaneous injection. After 7 days, mice were intravenously injected with 1×10^6 MSC-TERT-GRP78 (clone 4) loaded with Ad-3484-shHSP27-shTGF-β1. On the indicated days after intravenous injection, samples of tumor, liver, lung, spleen, kidney, and heart were collected, and total genomic DNA of each tissue was extracted. A standard curve was created using adenoviral DNA of known concentrations, and the standard curve was used to determine the viral load of each tissue sample. Black points represent individual mice (n = 4–5). (E) Measurement of luciferase activity in mouse tissues. Tumors, livers, and lungs were collected from mice in each group (n = 2–4) at 6 and 24 h after intravenous injection with Ad-Luc/MSC complex. After lysis of thawing frozen tissue powders and centrifugation, the supernatant was used for the detection of luciferase activity. Luciferase activities of tissues were calculated as ratios of firefly luciferase to Renilla luciferase activities, and the activities were reported as the ratios divided by the weight of each tissue.

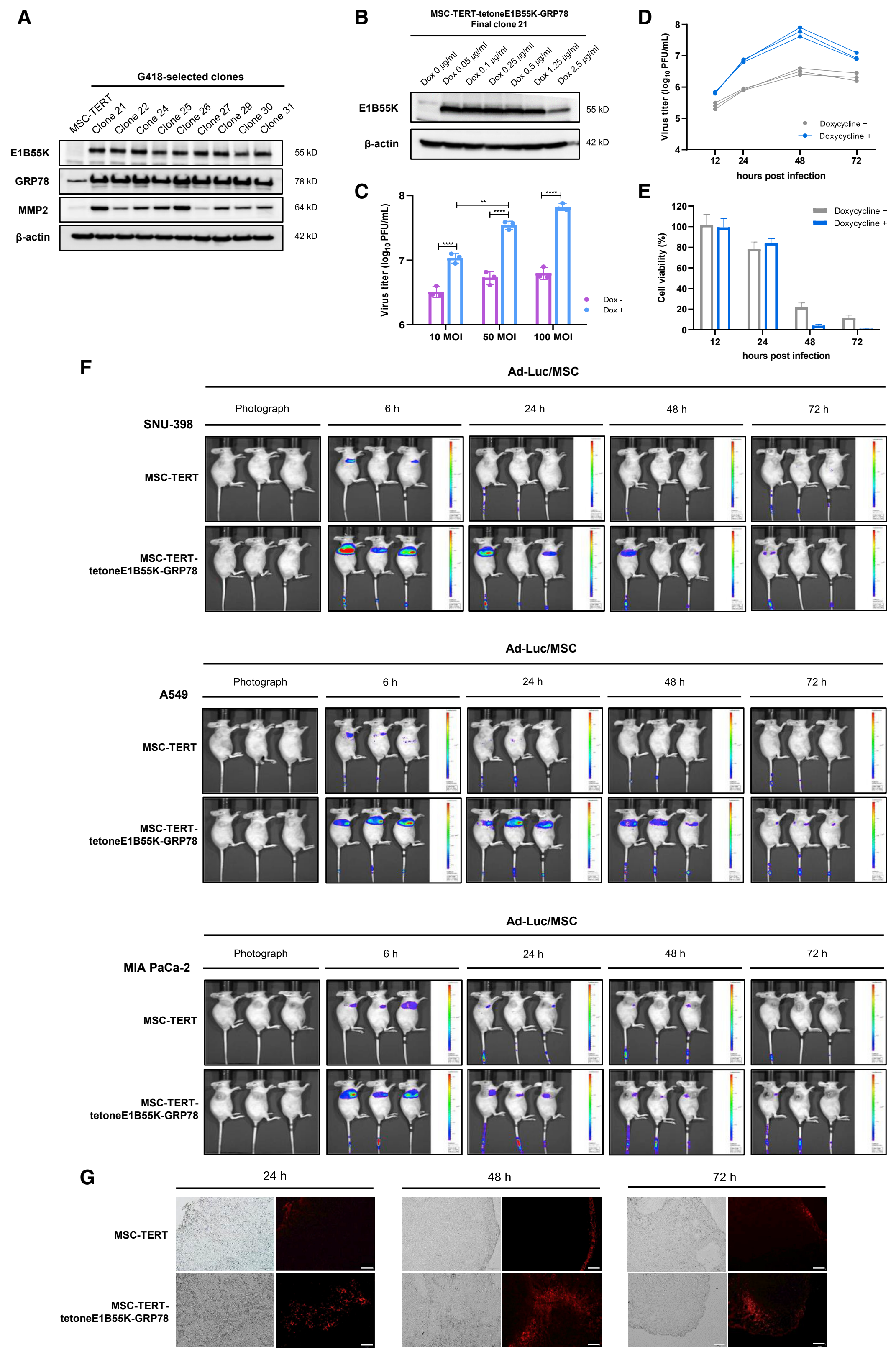

(legend on next page)

by shielding the virus from the immune system. Increasing the number of MSCs that reach the tumor and the overall tumor-targeting accuracy extends the utility of MSCs for tumor treatment.

Timely viral release of oncolytic adenoviruses from modified MSCs maximized the Trojan horse effect and was facilitated by a newly identified biological role for viral E1B55K, which induces apoptosis via p53 accumulation. The effect is the stoichiometrically opposite effect of its usual role in MSCs lacking E4orf6 expression. As a result, significantly enhanced tumor-targeting speed and increased viral release were observed, even in BALBc athymic mice, which retain innate immune responses associated with B and natural killer cells and are capable of viral clearance. This route to oncolysis achieved the goal of tumor regression without requiring adaptive T cell activation. Enhanced tumor cell killing was attributed, at least in part, to the expression of shRNAs targeting HSP27 and TGF-β1, which synergistically repressed tumor survival, reduced expression of tumor progression-related proteins, and concomitantly increased viral replication.

Surprisingly, the induction of E1B55K expression via the TetOne regulatory system was very highly regulated, with no expression observed in the absence of the inducer (doxycycline) and saturated expression induced by even the lowest doxycycline concentration examined. Integrating this regulatory system into MSCs facilitated the regulation of tumorigenic potential in passage-extended MSCs and facilitated the release of the oncolytic adenovirus after the arrival of MSCs at the tumor site, enhancing safety by minimizing the toxicity in other organs. No acute deaths were observed among more than 200 mice after tail vein injection of modified MSCs expressing oncolytic adenovirus.

Regulation of MSC tumorigenicity was also demonstrated experimentally. The tumor volumes in the untreated group and in mice treated with modified MSCs alone did not differ significantly, and no decrease in volume was observed for either group (Figures 7B–7D), suggesting that doxycycline-induced E1B55K expression in MSCs contributes to apoptotic death (Figure 8C). The possible removal of tumorigenic potential from the MSC-TERT-tetoneE1B55K-GRP78 clone was assessed by performing clonogenic assays, which showed that 14 days of doxycycline treatment effectively eliminated most MSCs (Figure 8C). Conclusively, we found that the E1B55K-induced death of MSCs and the introduction of the TetOne regulatory system practically eliminated the tumorigenic potential of MSCs. Moreover, the antitumor activity of a lower dose of MSCs was also evaluated, as the equivalent dose of 1 million MSCs per mouse, or 50 million MSCs per kilogram, would be an unrealistic cell number for delivery to a human patient. A reduced dose equal to 20% of the original (1×10^6 to 2×10^5 cells) continued to result in tumor regression (Figures 7F and 7G), demonstrating the potential clinical applicability of MSCs.

The results of this study demonstrate proof of concept for MSCs infected with oncolytic adenoviruses, which demonstrate efficient tumor targeting, regulated viral production, and *in vivo* antitumor efficacy. However, some limitations exist in our study. The complete suppression of tumor growth was not observed in some mice, even after the second intravenous injection of MSCs infected with oncolytic adenovirus (Figures 7B–7D), which may be due to limitations associated with our immune-deficient animal model (BALB/c athymic mice), such as the incomplete boosting of the antitumor immune system by the activation of the adaptive T cell response upon the oncolytic release of tumor antigens,[68] or to the rapid clearance of the oncolytic virus in the xenograft model by innate circulating factors.[63] A critical aspect of oncolytic virotherapy is maintaining a balance between the anti-viral and anticancer (cytotoxic T cell-mediated) immune responses.[68]

Aside from humanized mice, no mouse models used in studies of oncolytic adenovirus efficacy have been reported that exhibit a balance between these immune responses. Furthermore, no suitable immunocompetent mouse models (syngeneic type) are currently available due to minimal adenoviral replication in mice.[44,69,70] We anticipate that a greater therapeutic effect would be observed using humanized mice due to the activation of the adaptive T cell response by the oncolytic release of tumor antigens and the decreased immunosuppressive effect associated with shRNA repression of the target gene, TGF-β1.

Figure 6. Each function of MSC-TERT-tetoneE1B55K-GRP78 works well

(A) Expression levels of E1B55K, GRP78, and MMP2 in G418-selected clones in the presence of 0.5 μg/mL doxycycline. The final clone 21 stably overexpressed GRP78 and MMP2 and was selected as MSC-TERT-tetoneE1B55K-GRP78. (B) Induction of E1B55K expression in MSC-TERT-tetoneE1B55K-GRP78 in the presence of various concentrations of doxycycline. (C) Measuring virus production levels in MSC-TERT-tetoneE1B55K-GRP78. Cells were infected with indicated MOIs of Ad-3484-shHSP27-shTGF-β1 for 4 h and treated with or without doxycycline. After 48 h, all virus particles released from MSC-TERT-tetoneE1B55K-GRP78 were collected and the amount of infectious virus particles was calculated. Error bars are mean ± SD from three independent experiments: $^{**}p < 0.01$, $^{****}p < 0.0001$. (D) Quantification of viral production kinetics *in vitro* after infection of oncolytic virus to MSC-TERT-tetoneE1B55K-GRP78. Cells were infected with 100 MOIs of Ad-3484-shHSP27-shTGF-β1 for 4 h and treated with or without doxycycline. At the indicated time points, all virus particles released from MSC-TERT-tetoneE1B55K-GRP78 were collected and the amount of infectious virus particles was calculated. Data from three independent experiments are shown. (E) Cell viabilities of MSC-TERT-tetoneE1B55K-GRP78 loaded with oncolytic virus at the indicated time points were examined after infection with 100 MOIs of Ad-3484-shHSP27-shTGF-β1 for 4 h and treated with or without doxycycline. (F) Bioluminescence imaging of MSC-TERT or MSC-TERT-tetoneE1B55K-GRP78 loaded with Ad-Luc in mice bearing SNU-398 (upper), A549 (middle), or MIA PaCa-2 (lower) tumors. MSC-TERT and MSC-TERT-tetoneE1B55K-GRP78 distribution was monitored *in vivo* using the IVIS Spectrum System at 6, 24, 48, and 72 h after tail vein injection of Ad-Luc-loaded MSCs. The photo on the far left of each row shows the transplantation location of the tumor. Each scale bar shows intensity of bioluminescence; minimum and maximum radiances are indicated. (G) *In vivo* distribution of fluorescent probe-labeled MSCs. MSC-TERT or MSC-TERT-tetoneE1B55K-GRP78 was stained by cell tracker red fluorescent dye and intravenously injected into A549 tumor-bearing mice. At 24 h (upper), 48 h (middle), and 72 h (lower) after intravenous injection, tumor tissues were prepared by necropsy and frozen sectioned. All slides were observed under a fluorescence microscope with excitation at 540–585 nm and emission at 600 nm. Scale bars, 100 μm.

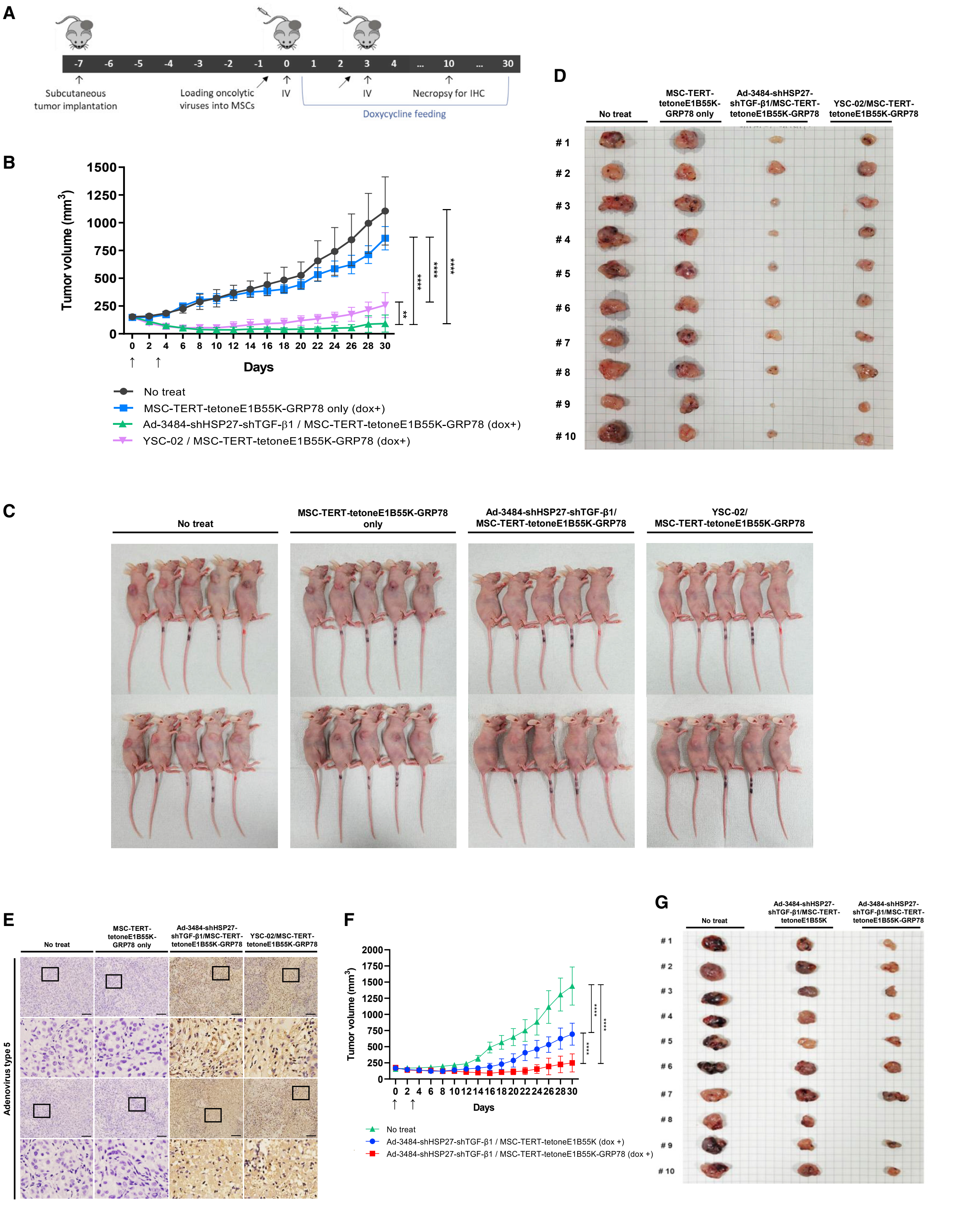

(legend on next page)

Our study significantly advances the tumor-targeting capability and efficacy of oncolytic adenoviruses. Because of their superior tumor-targeting abilities, modified MSCs infected with oncolytic adenovirus could also be useful for treating metastatic disease. Our results provide a sound basis for additional efforts to extend the clinical applicability of oncolytic adenovirus-based anticancer biologic treatment strategies.

MATERIALS AND METHODS

Cell lines

Human adipose tissue-derived MSCs and human bone marrow-derived MSCs were purchased from Cyagen Biosciences (China). MSCs were incubated at 37°C inside a 5% CO_2 humidified incubator using human mesenchymal stem cell growth medium (Cyagen Biosciences, China). A549, MDA-MB-231, MIA PaCa-2, A375, SK-BR-3, HPAC, HCT116, BJ, and HeLa cells were purchased from ATCC. SNU-398, DU145, Hep3B, and SK-HEP-1 were purchased from Korean Cell Line Bank and 293A cell (a subclone of HEK293) was purchased from Invitrogen (R70507). Cells were grown in Dulbecco's modified Eagle's medium supplemented with 10% fetal bovine serum (HyClone Laboratories, Logan, UT, United States) at 37°C under 5% CO_2.

Constructing replication-incompetent adenovirus expressing HSP27 and TGF-β1 shRNAs

For the expression of siRNA targeting human TGF-β1, the annealed oligonucleotide of shTGF-β1 was subcloned into *BamH*I, *Hind*III digested adenoviral E3 region shuttle vector,[35] pSP72ΔE3-U6. Then, to knock down both HSP27 and TGF-β1, U6-shTGF-β1 fragment after *Sph*I-blunt-*Kpn*I digested pSP72ΔE3-U6-shTGF-β1 was subcloned into *Hind*III-blunt-*Kpn*I digested pSP72ΔE3-H1-shHSP27, which was derived from pSP72ΔE3-H1-shDaxx.[39] The annealed insert sequences originated from Kim et al.[36] The resultant shuttle vector, including two shRNAs, pSP72ΔE3-H1-shHSP27-U6-shTGF-β1, was linearized by *Xmn*I digestion, and the adenoviral backbone vector, dl324-IX, was linearized by *Spe*I digestion. The linearized vectors were cotransformed into *Escherichia coli* BJ5183 competent cells for homologous recombination. The final adenoviral construct, dl324-IX-H1-shHSP27-U6-shTGF-β1, was digested with *Pac*I and transfected into 293A cells to generate replication-incompetent adenovirus expressing shRNAs targeting HSP27 and TGF-β1.

Constructing oncolytic adenovirus expressing HSP27 and TGF-β1 shRNAs

The adenoviral E3 region shuttle vector, pSP72ΔE3-H1-shHSP27-U6-shTGF-β1, was linearized by *Xmn*I digestion, and the adenoviral backbone vector, dl324-BstBI, was linearized by *Spe*I digestion. The two linearized vectors were cotransformed into *E. coli* BJ5183 competent cells for the first homologous recombination. Then, a second homologous recombination was performed using dl324-BstBI-H1-shHSP27-U6-shTGF-β1 and the adenoviral shuttle vector pVAX1-3484, which contains an E1 region without E1B55K, to generate E1B55K-deleted oncolytic adenovirus arming HSP27 and TGF-β1 shRNAs. The final adenoviral construct, dl324-3484-H1-shHSP27-U6-shTGF-β1, was digested with *Pac*I and transfected into 293A cells to generate the tumor-selective replicative adenovirus. The 293A cells were reinfected with the generated adenovirus, and this procedure was repeated for viral amplification. The infectious titer of the adenovirus was determined by performing a limiting dilution assay in 293A cells after concentration and purification with CsCl gradients.

Constructing replication-incompetent adenovirus expressing RFP

The RFP gene was subcloned from *Nru*I, *Xba*I-digested pcDNA3-mRFP (Addgene, #13032) into the adenoviral E1 region shuttle vector *Sna*BI, *Xba*I-digested pCA14. The resulting adenoviral shuttle vector, pCA14-RFP, was linearized by *Xmn*I digestion. The adenoviral vector dl324-BstBI was linearized by *Bsp119*I digestion, and the two linearized vectors were cotransformed into *E. coli* BJ5183 competent cells for homologous recombination.

Constructing replication-competent adenovirus expressing RFP

The RFP gene was subcloned from pcDNA3-mRFP into the adenoviral E1 region shuttle vector pVAX1-3484. Two PCR primers containing the *Sal*I restriction were used to amplify the RFP coding sequence, which was then ligated with *Sal*I-digested pVAX1-3484. The primers used for amplification are listed in Table S1. RFP gene insertion into the pVAX1-3484 shuttle vector was confirmed by analyzing the RFP expression level in 293A cells transfected with positive control pcDNA3-mRFP. The adenoviral shuttle vector, pVAX1-3484-RFP, was linearized by *Pme*I digestion. The adenoviral vector dl324-BstBI was linearized by *Bsp119*1 digestion, and the two

Figure 7. MSC-TERT-tetoneE1B55K-GRP78 significantly enhances *in vivo* antitumor efficacy of oncolytic adenovirus

(A) Schematic overview of the animal study procedure. (B) Tumor volume measurement in A549 xenograft models. Tumor cells were implanted subcutaneously in the flank region of BALB/c athymic nude mice, and 1×10^6 MSC-TERT-tetoneE1B55K-GRP78 or MSC-TERT-tetoneE1B55K-GRP78 loaded with oncolytic adenovirus was injected intravenously twice with an interval of 3 days. Tumor volume was monitored and recorded every 2 days until the end of the study. Arrows indicate when the MSC or oncolytic adenovirus/MSC complex was administered. Error bars represent mean ± SD from 10 mice per group: **$p < 0.01$, ****$p < 0.0001$. (C and D) All mice in each experimental group (C) and tumor tissues of mice obtained by necropsy (D) at the end of the experiment. Every mouse was individually tracked. (E) The expression of adenovirus type 5 structural protein in tumor tissues was analyzed by immunohistochemistry at 7 days after the second intravenous injection. Representative images show the average of all slides for each group. Rows 2 and 4 are enlarged images of the black squares in rows 1 and 3. Scale bars, 100 μm. (F) Tumor volume measurements in A549 xenograft models using lower number of modified MSCs. Tumor cells were implanted subcutaneously in the flank region of BALB/c athymic nude mice, and 2×10^5 MSC-TERT-tetoneE1B55K or MSC-TERT-tetoneE1B55K-GRP78 loaded with oncolytic adenovirus (Ad-3484-shHSP27-shTGF-β1) was injected intravenously twice with an interval of 3 days. Tumor volume was monitored for 30 days and recorded every 2 days. Error bars represent mean ± SD from 10 mice per group. (G) All tumor tissues of mice in each experimental group (no treatment, Ad-3484-shHSP27-shTGF-β1/MSC-TERT-tetoneE1B55K, and Ad-3484-shHSP27-shTGF-β1/MSC-TERT-tetoneE1B55K-GRP78) were obtained by necropsy at the end of the experiment. Every mouse was individually tracked.

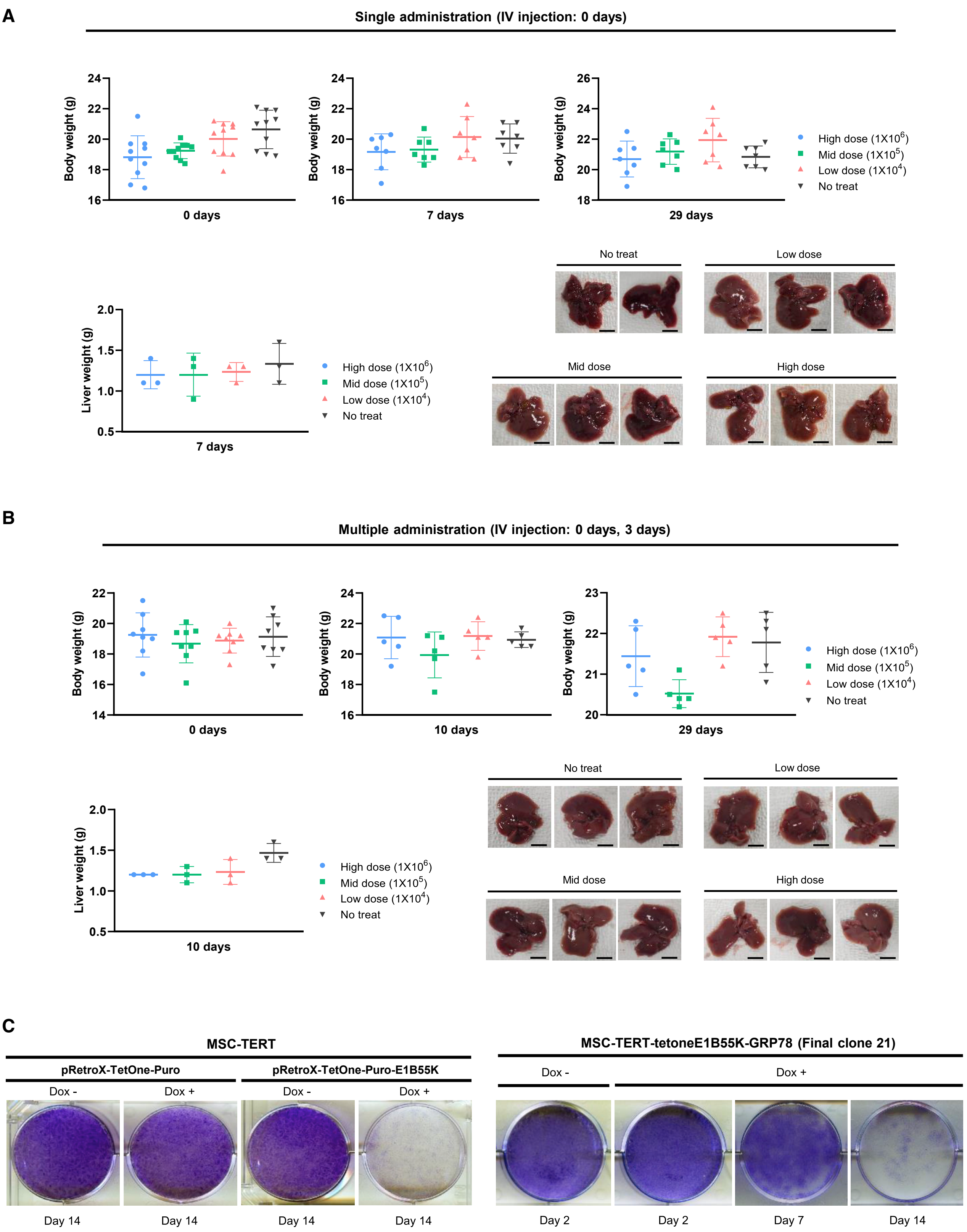

(legend on next page)

linearized vectors were cotransformed into *E. coli* BJ5183 competent cells for homologous recombination.

Subcloning E1B55K into the expression vector

The E1B55K gene was subcloned from pBSK-3484 into the pcDNA3.1-Hygro vector (Invitrogen, United States). Two PCR primers containing the *BamH*I and the *Not*I restriction sites were used to amplify the E1B55K coding sequence from pBSK-3484. The resulting product was ligated with the *BamH*I, *Not*I digested pcDNA3.1-Hygro vector. The primers used for amplification are listed in Table S1.

Subcloning of E4orf6 into the expression vector

The E4orf6 (E434K) gene was subcloned from dl324-BstBI into the pFlag-CMV2 vector (Sigma-Aldrich, United States). Two PCR primers containing the *Hind*III and *Xba*I restriction sites were used to amplify the E4orf6 coding sequence from dl324-BstBI. The primers used for amplification are listed in Table S1. The resulting product was ligated with the *Hind*III, *Xba*I-digested pFLAG-CMV2 vector.

Generating E1B55K and E4orf6 polyclonal antibodies

Human adenovirus type 5 E1B55K and E4orf6 proteins (GenBank : AC_000008.1) were detected using polyclonal antibodies. The E1B55K and E4orf6 antibodies were raised against synthetic peptides from the N terminus of E1B55K (MERRNPSERGVPAGFSGHAS VESGC) or the N terminus of E4orf6 (RPTRSRLSRRTPYSRDRL PPFE). The synthetic peptides were selected from five or four candidates, respectively, and then conjugated to bovine serum albumin (BSA) as an immunogenic carrier protein. These products were used to immunize two New Zealand white rabbits. All of these procedures, including antibody production and purification, were performed by GW Vitek (Korea).

Microarray analysis

Total RNA was extracted and purified using the RNeasy Plus Mini Kit (Qiagen, 74134) according to the manufacturer's instructions. The results were summarized and normalized by Affymetrix Power Tools (APT) or Illumina GenomeStudio v2011.1. These data were exported for differentially expressed gene (DEG) analysis. The statistical significance of the expression data was determined using fold change. Hierarchical cluster analysis was performed for a DEG set using complete linkage and Euclidean distance as a measure of similarity. All data analysis and visualization of DEGs was conducted by Macrogen (Korea) using R 3.3.2.

Clonogenic assays

Cells were seeded at 2×10^5 cells per well in six-well plates, and then infected with replication-incompetent adenovirus the next day. After incubation for 48 h, the cells were redistributed in six-well plates at dilution factors of 1/200, 1/100, and 1/50, and incubated for an additional 7–14 days. These plates were checked daily under a microscope. When the cells formed several colonies, they were fixed with 4% paraformaldehyde and stained with 0.5% crystal violet.

Luciferase reporter assay

MSC-TERT-tetoneE1B55K cells were transfected with pGL2-356bp (Addgene, #16292), and then treated with doxycycline for 48 h to induce E1B55K expression. The transcriptional activity of p53 was determined using the dual-luciferase reporter assay system according to the manufacturer's instructions (Promega, E1910). The p53 promoter activity was calculated by measuring the firefly luciferase activity.

Constructing replication-incompetent adenovirus expressing E1B55K

For cloning, the E1B55K gene of the adenoviral genome was subcloned from pBSK-3484 into the adenoviral E1 region shuttle vector pCA14. Two PCR primers containing the *Xba*I and *Hind*III restriction sites were used to amplify the E1B55K coding sequence and are listed in Table S1. Then, the product was ligated with *Xba*I, *Hind*III digested pCA14. The resulting adenoviral shuttle vector, pCA14-E1B55K, was linearized by *Xmn*I digestion, and the adenoviral vector dl324-BstBI was linearized by *Bsp119*I digestion. The two linearized vectors were cotransformed into *E. coli* BJ5183 competent cells for homologous recombination.

Cloning GRP78 from SNU-449 into the pcDNA3.1-Hygro expression plasmid

Total RNA was extracted from SNU-449, a human hepatocellular carcinoma cell line with high GRP78 expression levels (purchased from the Korea Cell Line Bank), using TRIzol reagent (Invitrogen). The extracted RNA was then reverse transcribed using the SuperScript First-Strand Synthesis System for RT-PCR kit (Invitrogen), followed by PCR. An *Xho*I site was added at the 5′ end of the forward primer, and an *Xba*I site was added at the 3′ end of the reverse primer. The primers used for amplification are listed in Table S1. The resultant PCR product was digested with *Xho*I/*Xba*I, and then ligated into *Xho*I/*Xba*I-digested pcDNA3.1-Hygro vector. The GRP78 coding sequence inserted into the pcDNA3.1-Hygro vector was confirmed

Figure 8. Toxicity study and schematic diagram of cancer gene therapy using Ad-3484-shHSP27-shTGF-β1/modified MSC complex

(A and B) BALB/c athymic nude mice were divided into four groups with 8–10 mice in each. The Ad-3484-shHSP27-shTGF-β1/modified MSC complex was administered intravenously as a single (A) or multiple injection (B) at different dose levels of 1×10^4 (low dose), 1×10^5 (middle dose), and 1×10^6 (high dose) cells. Body weight of each mouse was graphed at 0, 7, and 29 days in single administration and 0, 10, and 29 days in multiple administration. At 7 days after the last intravenous injection, three mice were necropsied to check the liver condition. Scale bars, 50 mm. (C) 1×10^5 cells of MSC-TERT were seeded on a six-well plate and transfected with 1 μg of pRetroX-TetOne-Puro or pRetroX-TetOne-Puro-E1B55K. The next day, cells were treated with or without doxycycline and redistributed 48–72 h after treatment. Cells were incubated for an additional 14 days with doxycycline treatment every other day. At the indicated time point, cells were fixed and stained (left). 1×10^5 cells of MSC-TERT-tetoneE1B55K-GRP78 were seeded on a six-well plate and treated with or without doxycycline the next day. From 48 to 72 h after treatment, cells were redistributed and incubated for an additional 2–14 days with doxycycline treatment every other day. At the indicated time point, cells were fixed and stained (right).

to be consistent with the NCBI reference sequence GenBank : NM_005347.5.

Generating modified MSCs

Platinum-A packaging cells (Cell Biolabs, United States) were transfected with the retroviral expression vector pBABE-hygro-hTERT (Addgene, #1773). After 48 h, the packaging cell culture medium was collected and filtered through a 0.2-μm cellulose acetate filter. Human adipose tissue-derived MSCs were infected with the filtered retroviral supernatant and cultured in the selection medium containing 200 μg/mL hygromycin B gold (InvivoGen, United States). Individual hygromycin-resistant colonies were expanded and screened for TERT-positive expression using western blots. The best cell clone (clone 22) was selected and expanded for use in subsequent experiments. Subsequently, the E1B55K gene was subcloned from pCA14-E1B55K into the Retro-X-TetOne inducible expression system (TAKARA Bio, Japan) using the In-Fusion HD Cloning kit (TAKARA Bio). E1B55K was amplified using the in-fusion *Eco*RI forward primer and the in-fusion *Bam*HI reverse primer. The primers used for amplification are listed in Table S1. For the production of retrovirus, pRetro-X-TetOne-Puro-E1B55K vector was cotransfected with the pAmpho envelope vector into the GP2-293 packaging cells. After 48 h, MSC-TERT (clone 22) was infected with the filtered retroviral supernatant in the presence of polybrene. Cells were cultured in the selection medium containing 0.8–1.0 μg/mL puromycin (InvivoGen), and every collected puromycin-resistant colony was verified for inducible expression of E1B55K by western blotting in the presence of doxycycline (0.05–2.5 μg/mL).

Finally, retrovirus was produced in Platinum-A cells using the retroviral expression vector pLNCX neo-GRP78. pLNCXneo was designed for the extension of cloning sites from HindIII-HpaI-ClaI of pLNCX to HindIII-PmlI-BstXI-NotI-XhoI-SalI-ApaI-PmeI-HpaI-ClaI, and GRP78 from pcDNA3.1-GRP78 after digestion with XhoI/PmeI was subcloned into XhoI/PmeI-predigested pLNCXneo. Then, the virus produced from transfection of pLNCXneo-GRP78 to Platinum-A cells was used to infect MSC-TERT-tetoneE1B55K (clone 2) as stated above. Cells were cultured in medium containing 500–600 μg/mL G418 (InvivoGen) for selecting stable clones. The clone stably overexpressing GRP78 and MMP2 was identified by western blot. This final modified human MSC clone, MSC-TERT-tetoneE1B55K-GRP78, was used for *in vitro* and *in vivo* studies. The MSC-TERT-GRP78 clones were also generated using the same methods as described above.

Quantifying infectious virus particles

The quantity of infectious virus particles in the adenoviral supernatant was calculated using three cycles of freezing and thawing the infected cells and supernatant, followed by centrifugation at 3,500 rpm for 10 min. The adenoviral supernatant was serially diluted in the range of 10^{-1} to 10^{-8}, and 100 μL of each diluted supernatant was added to 1×10^4 293A packaging cells. After 7 days of incubation at 37°C, the number of wells exhibiting cytopathic effects in 293A cells was counted for the calculation. The number of infectious virus particles was calculated using the median tissue culture infectious dose (TCID50) and the following formula: titer (TCID50/mL) = $10^{[1+1(\text{counted wells}-0.5)]}$.

TCID50/mL was transformed into plaque-forming units (PFU) per milliliter by computing titer (PFU/mL) = $1 \times 10^{(\text{TCID50/mL}-0.7)}$.

Western blotting and antibodies

Cells were lysed in 1× Laemmli lysis buffer (62.5 mM Tris pH 6.8, 2% sodium dodecyl sulfate, 10% glycerol, 0.02% bromophenol blue). Proteins were separated by sodium dodecyl sulfate-polyacrylamide gel electrophoresis (SDS-PAGE) and transferred by electroblot onto polyvinylidene fluoride (PVDF) membranes (Millipore, United States). Each PVDF membrane was blocked with 5% non-fat dry milk or 5% BSA in Tris-buffered saline (TBS) Tween 20 (0.1%, v/v) buffer at room temperature for 1 h. Then, the membranes were incubated with the primary antibodies against HSP27(Enzo Life Sciences, United States), N-cadherin, β-catenin, Daxx, Vimentin, Akt, MMP2, p53, Mdm2 (Cell Signaling Technology, United States), β-actin, GAPDH, TERT, MMP1, CXCR4 (Santa Cruz, United States), CD44 (Invitrogen, United States), or GRP78 (Proteintech, United States) for 1 h to overnight depending on the antibodies. Horseradish peroxidase (HRP)-conjugated anti-mouse or anti-rabbit immunoglobulin (Ig) G was used as a secondary antibody. Immunoreactive proteins were visualized using an enhanced chemiluminescence (ECL) substrate (Thermo Fisher Scientific, 32106).

Constructing replication-incompetent adenovirus expressing firefly luciferase

The firefly luciferase gene was subcloned from pLXIN-Luc (Addgene, #60683) into the adenoviral E1 region shuttle vector pCA14 using *Eco*RI digestion. The adenoviral shuttle vector, pCA14-Luc, was linearized by *Xmn*I digestion, and the adenoviral vector dl324-BstBI was linearized by *Bsp119*I digestion. The two linearized vectors were cotransformed into *E. coli* BJ5183 competent cells for homologous recombination.

Animal studies for comparative analyses of antitumor efficacy

Animal studies were conducted in compliance with the ARRIVE guidelines following a protocol approved by Yonsei University Health System's Institutional Animal Care and Use Committee (IACUC) and consistent with the guidelines of the Animal Welfare Act and the Guide for the Care and Use of Laboratory Animals. Female BALB/cSlc-*nu/nu* mice weighing 12–20 g were obtained from Japan SLC. All mice were housed in specific pathogen-free facilities for a 1-week period of acclimation, received free feeding with a standard diet (PicoLab Rodent diet 20 5053) or doxycycline diet (Envigo, United States) and reverse-osmosis-purified water, in individually ventilated cages at a temperature of 21°C ± 0.2°C, humidity 50% ± 10%, and a 12/12-h light/dark cycle. The maximum caging density was five mice, and the bedding was autoclaved aspen chips.

For direct intratumoral injection studies, 5×10^6 A549 cells or 2×10^6 SNU-398 cells were implanted with Matrigel matrix into

the subcutaneous right flank region of 4- to 6-week-old BALB/c athymic nude mice. When the tumors reached an average volume of 80–110 mm^3, 1×10^9 PFU of each oncolytic adenovirus diluted in 50 μL of PBS or 50 μL of PBS alone was injected directly into tumors. Intratumoral injections were performed twice at an interval of 3 days. Tumor growth or regression was assessed by measuring the tumor length (L) and width (W). The tumor volume was calculated using the following formula: volume (mm^3) = $0.52 \times L \times W^2$.

For systemic intravenous injection studies, 8×10^6 A549 cells were implanted with Matrigel matrix into the subcutaneous right flank region of 4- to 6-week-old BALB/c athymic nude mice. When the tumors reached an average volume of 150 mm^3, the mice received one of the following intravenous injections: 1×10^6 or 2×10^5 modified MSCs only, modified MSCs loaded with oncolytic adenovirus expressing shHSP27 and shTGF-β1, or modified MSCs loaded with oncolytic adenovirus expressing human GM-CSF and Flt3L-TRAIL, in addition to shHSP27 and shTGF-β1 (named YSC-02) suspended in 100 μL OF PBS with five units of heparin per mouse. Modified MSCs alone or loaded with oncolytic adenovirus were administered twice at intervals of 3 days. Doxycycline was given in their feed at a concentration of 625 mg/kg from immediately after the intravenous administration at the end of the experiment. The tumors were measured using electronic calipers, and tumor volume was calculated using the following formula: volume (mm^3) = $0.52 \times L \times W^2$.

Bioluminescence imaging in tumor-bearing mice

Before imaging, 4- to 6-week-old BALB/c athymic nude mice were implanted with 8×10^6 A549 cells, 2×10^6 SNU-398 cells, or 2×10^6 MIA PaCa-2 cells by subcutaneous injection at the right shoulder. After 7–14 days, the mice received intravenous injection with 1×10^6 MSC-TERT, MSC-TERT-GRP78, or MSC-TERT-tetoneE1B55K-GRP78 loaded with replication-incompetent adenovirus expressing firefly luciferase. For luminescence measurements, animals were injected intraperitoneally with 150 mg/kg D-luciferin (PerkinElmer, United States). The distribution of MSCs *in vivo* was monitored using the IVIS Spectrum System (PerkinElmer) at 6, 24, 48, and 72 h after intravenous injection of luciferase-expressing adenoviruses loaded into three types of MSC.

Measurement of luciferase activity in mouse tissue

Tumors, livers, and lungs were collected from mice in each group (n = 2–4) at 6 and 24 h after intravenous injection of the Ad-Luc/MSC complex. Tissues were frozen on dry ice immediately after collection and stored at −80°C. Frozen tissues were individually pulverized into a fine powder by hand grinding with a chilled porcelain mortar and pestle and the powder was stored at −80°C until extraction. Frozen tissue powders were thawed and 500 μL of Promega lysis buffer (E194A) was added to each sample. The samples were vortexed for 15 min, then frozen and thawed three times using alternating liquid nitrogen and 37°C water baths. The sample was then centrifuged for 3 min at 10,000 × *g*, and the supernatant was transferred to a new tube. The extraction process was repeated without freeze thawing after adding another 500 μL of lysis buffer to the pellet. The second supernatant was combined with the first one and the nearly 1 mL of extract was stored at −80°C until used in an assay. Luciferase activities were determined using the dual-luciferase reporter assay system according to the manufacturer's instructions (Promega, E1910). Luciferase activities of tissues were calculated as ratios of firefly luciferase to Renilla luciferase activities and were reported as the ratios divided by the weight of each tissue.

Real-time qRT-PCR

The mRNA expression patterns of adenovirus expressing shRNAs were detected as follows. Cells were infected with each adenovirus as described above or transfected with expression vectors, and then total RNA was extracted using TRIzol reagent (Invitrogen). The RNA was reverse transcribed into cDNA using ReverTra Ace qPCR RT Master Mix (Toyobo, Japan), and qPCR was performed using Power SYBR Green PCR Master Mix (Thermo Fisher Scientific, 4367659). The primers used for quantification are listed in Table S2.

qPCR was performed to calculate adenovirus copy numbers in tissues as follows: 4- to 6-week-old BALB/c athymic nude mice were transplanted with 2×10^6 SNU-398 cells by subcutaneous injection at the right flank region. After 7 days, the mice (n = 4–5 per day) received intravenous injection with 1×10^6 MSC-TERT-GRP78 loaded with oncolytic adenovirus. Samples of tumor, liver, lung, spleen, kidney, and heart were collected at 1, 2, 3, 4, 5, 10, and 15 days after the intravenous injection, and total genomic DNA of each tissue was extracted using the DNeasy Blood & Tissue Kit (Qiagen, 69506). Local variation for each tissue was minimized by homogenizing all tissues into powder. The qRT-PCR assays were performed using TB Green Premix Ex Taq (Takara, RR402A), with at least two repeated experiments per sample, and the average value was used as the result. A standard curve was prepared using known concentrations of adenoviral DNA and the standard curve was used to determine the viral load in each tissue sample. The primers used for quantification were adenoviral serotype 5 E4orfB region primers and are also listed in Table S2. Adenovirus copy numbers were calculated using a formula that requires the amount of DNA and the length of template considering molecular weight: number of copies = $(ng \times 6.022 \times 10^{23})/(length \times 1 \times 10^9 \times 660)$.

Cell tracker labeling

The 4- to 6-week-old BALB/c athymic nude mice were implanted with 8×10^6 A549 cells by subcutaneous injection at the right flank region. After 7 days, the mice received intravenous injection with 1×10^6 cell tracker red fluorescent probe (Invitrogen, C34565)-labeled modified MSCs. At 24, 48, and 72 h after intravenous injection, each tumor tissue was obtained by necropsy and frozen sectioned. All slides were observed using a fluorescence microscope with 540–585-nm excitation and 600-nm emission.

Immunohistochemistry

Tumor tissues of two mice per group were obtained by necropsy at 7 days after the second intravenous injection. All tissues were fixed for at least 24 h in 10% formaldehyde, embedded in paraffin, and

then cut into 40-μm sections. Tissue section slides were deparaffinized with xylene twice for 10 min, and the slides were rehydrated using a graded alcohol series. Following antigen retrieval, endogenous peroxidase from each tissue section was removed using 3% hydrogen peroxide. After blocking for 1 h with 5% BSA, slides were incubated with anti-adenovirus type 5 antibody (Abcam, UK) overnight at 4°C to detect adenovirus structural proteins (e.g., hexon, penton, and fiber). Horseradish peroxidase (HRP)-conjugated anti-rabbit IgG was used as a secondary antibody and incubated for 1 h at room temperature. To detect the chromogenic activity of HRP, a mixture of 3,3′-diaminobenzidine tetrahydrochloride (DAB) substrate (Thermo Fisher Scientific, 34002) was added for 5–10 min. After washing with distilled water, hematoxylin (Dako, Denmark) was added for 2–5 min to stain the nuclei. The dehydrated and cleared tissue sections were mounted with mounting media (xylene:mount = 1:1) for microscopy.

Toxicity study

Toxicity studies were conducted on 5-week-old BALB/c athymic nude mice. All the animals were divided into four groups with 10 mice each. The oncolytic adenovirus/modified MSC complex was administered intravenously as a single or multiple injections at different dose of 1×10^4 (low dose), 1×10^5 (middle dose), and 1×10^6 (high dose) cells. Animals were observed for toxicity symptoms of body weight, food and water intake, and survival rate daily for 30 days. At 7 days after the last intravenous injection, three mice were necropsied to check their liver condition.

Statistical analysis

The results were presented as the mean ± standard deviation (SD). Differences between groups were examined using two-tailed Student's t tests or two-way analysis of variance (ANOVA). p values were calculated using GraphPad Prism 8.0. $p < 0.0001$, $p < 0.001$, $p < 0.01$, and $p < 0.05$ were considered as statistically significant.

DATA AND CODE AVAILABILITY

The authors confirm that the data supporting the findings of this study are available within the article and its supplementary figures. The microarray data have been deposited in the NCBI GEO database (GSE196290; GSE196290 is composed of the GSE196286, GSE196287, GSE196288, and GSE196289). Raw data that support the findings of this study are available from the corresponding author (J.J.S.), upon reasonable request.

SUPPLEMENTAL INFORMATION

Supplemental information can be found online at https://doi.org/10.1016/j.omto.2022.09.004.

ACKNOWLEDGMENTS

We thank Medical Illustration and Design, part of the Medical Research Support Services of Yonsei University College of Medicine, for all artistic support related to this work. This research was supported by the Bio and Medical Technology Development Program of the National Research Foundation (NRF) and funded by the Korean government (Ministry of Science and ICT [MSIT]) (NRF-2019M3E5D5064554). This work was also supported by a National Research Foundation of Korea (NRF) grant funded by the Korea government (MSIT) (NRF-2020R1A2C1005245). This research was also supported by the Korea Drug Development Fund funded by MSIT; Ministry of Trade, Industry, and Energy; and Ministry of Health and Welfare (HN21C0960, Republic of Korea). Finally, this study was also supported by a faculty research grant from Yonsei University College of Medicine (6-2021-0079).

AUTHOR CONTRIBUTIONS

S.C. and J.J.S. designed, analyzed the experiments, and wrote the manuscript. J.A.H. performed acquisition and curation of data. H.J.C. and J.J.S. conceived and supervised the study. All authors have read and approved the article.

DECLARATION OF INTERESTS

The authors declare that no competing interests.

REFERENCES

1. Russell, S.J., Peng, K.W., and Bell, J.C. (2012). Oncolytic virotherapy. Nat. Biotechnol. *30*, 658–670.
2. Moreno, R. (2021). Mesenchymal stem cells and oncolytic viruses: joining forces against cancer. J. Immunother. Cancer *9*, e001684.
3. Zheng, M., Huang, J., Tong, A., and Yang, H. (2019). Oncolytic viruses for cancer therapy: barriers and recent advances. Mol. Ther. Oncolytics *15*, 234–247.
4. Vähä-Koskela, M., and Hinkkanen, A. (2014). Tumor restrictions to oncolytic virus. Biomedicines *2*, 163–194.
5. Pilankatta, R., Chawla, T., Khanna, N., and Swaminathan, S. (2010). The prevalence of antibodies to adenovirus serotype 5 in an adult Indian population and implications for adenovirus vector vaccines. J. Med. Virol. *82*, 407–414.
6. Sun, C., Zhang, Y., Feng, L., Pan, W., Zhang, M., Hong, Z., Ma, X., Chen, X., and Chen, L. (2011). Epidemiology of adenovirus type 5 neutralizing antibodies in healthy people and AIDS patients in Guangzhou, southern China. Vaccine *29*, 3837–3841.
7. Barry, M.A., Rubin, J.D., and Lu, S.C. (2020). Retargeting adenoviruses for therapeutic applications and vaccines. FEBS Lett. *594*, 1918–1946.
8. Power, A.T., and Bell, J.C. (2008). Taming the Trojan horse: optimizing dynamic carrier cell/oncolytic virus systems for cancer biotherapy. Gene Ther. *15*, 772–779.
9. De Silva, N., Atkins, H., Kirn, D.H., Bell, J.C., and Breitbach, C.J. (2010). Double trouble for tumours: exploiting the tumour microenvironment to enhance anticancer effect of oncolytic viruses. Cytokine Growth Factor Rev. *21*, 135–141.
10. Xia, X., Ji, T., Chen, P., Li, X., Fang, Y., Gao, Q., Liao, S., You, L., Xu, H., Ma, Q., et al. (2011). Mesenchymal stem cells as carriers and amplifiers in CRAd delivery to tumors. Mol. Cancer *10*, 134–145.
11. Uccelli, A., Moretta, L., and Pistoia, V. (2008). Mesenchymal stem cells in health and disease. Nat. Rev. Immunol. *8*, 726–736.
12. Nakashima, H., Kaur, B., and Chiocca, E.A. (2010). Directing systemic oncolytic viral delivery to tumors via carrier cells. Cytokine Growth Factor Rev. *21*, 119–126.
13. De Becker, A., and Riet, I.V. (2016). Homing and migration of mesenchymal stromal cells: how to improve the efficacy of cell therapy? World J. Stem Cells *8*, 73–87.
14. Ullah, M., Liu, D.D., and Thakor, A.S. (2019). Mesenchymal stromal cell homing: mechanisms and strategies for improvement. iScience *15*, 421–438.
15. Lee, A.S. (2014). Glucose-regulated proteins in cancer: molecular mechanisms and therapeutic potential. Nat. Rev. Cancer *14*, 263–276.
16. Li, Z., Zhang, L., Li, H., Shan, S., and Li, Z. (2014). Glucose regulated protein 78 promotes cell invasion via regulation of uPA production and secretion in colon cancer cells. BMB Rep. *47*, 445–450.

17. Hidalgo, P., Ip, W.H., Dobner, T., and Gonzalez, R.A. (2019). The biology of the adenovirus E1B 55K protein. FEBS Lett. *593*, 3504–3517.

18. Kindsmüller, K., Schreiner, S., Leinenkugel, F., Groitl, P., Kremmer, E., and Dobner, T. (2009). A 49-kilodalton isoform of the adenovirus type 5 early region 1B 55-kilodalton protein is sufficient to support virus replication. J. Virol. *83*, 9045–9056.

19. Schreiner, S., Wimmer, P., Sirma, H., Everett, R.D., Blanchette, P., Groitl, P., and Dobner, T. (2010). Proteasome-dependent degradation of Daxx by the viral E1B-55K protein in human adenovirus-infected cells. J. Virol. *84*, 7029–7038.

20. Neuzillet, C., Tijeras-Raballand, A., Cohen, R., Cros, J., Faivre, S., Raymond, E., and de Gramont, A. (2015). Targeting the TGFβ pathway for cancer therapy. Pharmacol. Ther. *147*, 22–31.

21. Liu, S., Ren, J., and Ten Dijke, P. (2021). Targeting TGFbeta signal transduction for cancer therapy. Signal Transduct. Target. Ther. *6*, 8–27.

22. Neuzillet, C., de Gramont, A., Tijeras-Raballand, A., de Mestier, L., Cros, J., Faivre, S., and Raymond, E. (2014). Perspectives of TGF-β inhibition in pancreatic and hepatocellular carcinomas. Oncotarget *5*, 78–94.

23. Taylor, M.A., Lee, Y.-H., and Schiemann, W.P. (2011). Role of TGF-β and the tumor microenvironment during mammary tumorigenesis. Gene Expr. *15*, 117–132.

24. Wrzesinski, S.H., Wan, Y.Y., and Flavell, R.A. (2007). Transforming growth factor-β and the immune response: implications for anticancer therapy. Clin. Cancer Res. *13*, 5262–5270.

25. Akhurst, R.J., and Hata, A. (2012). Targeting the TGFβ signalling pathway in disease. Nat. Rev. Drug Discov. *11*, 790–811.

26. Tang, B., Yoo, N., Vu, M., Mamura, M., Nam, J.-S., Ooshima, A., Du, Z., Desprez, P.-Y., Anver, M.R., Michalowska, a.m., et al. (2007). Transforming growth factor-β can suppress tumorigenesis through effects on the putative cancer stem or early progenitor cell and committed progeny in a breast cancer xenograft model. Cancer Res. *67*, 8643–8652.

27. Syed, V. (2016). TGF-β signaling in cancer. J. Cell. Biochem. *117*, 1279–1287.

28. Jolly, C., and Morimoto, R.I. (2000). Role of the heat shock response and molecular chaperones in oncogenesis and cell death. J. Natl. Cancer Inst. *92*, 1564–1572.

29. Cheng, J., Lv, Z., Weng, X., Ye, S., Shen, K., Li, M., Qin, Y., Hu, C., Zhang, C., Wu, J., and Zheng, S. (2015). Hsp27 acts as a master molecular chaperone and plays an essential role in hepatocellular carcinoma progression. Digestion *92*, 192–202.

30. Vidyasagar, A., Wilson, N.A., and Djamali, A. (2012). Heat shock protein 27 (HSP27): biomarker of disease and therapeutic target. Fibrogenesis Tissue Repair *5*, 7–13.

31. Kang, D., Choi, H.J., Kang, S., Kim, S.Y., Hwang, Y.S., Je, S., Han, Z., Kim, J.H., and Song, J.J. (2015). Ratio of phosphorylated HSP27 to nonphosphorylated HSP27 biphasically acts as a determinant of cellular fate in gemcitabine-resistant pancreatic cancer cells. Cell. Signal. *27*, 807–817.

32. Arrigo, A.P., and Gibert, B. (2014). HspB1, HspB5 and HspB4 in human cancers: potent oncogenic role of some of their client proteins. Cancers *6*, 333–365.

33. Han, Z., Kang, D., Joo, Y., Lee, J., Oh, G.-H., Choi, S., Ko, S., Je, S., Choi, H.J., and Song, J.J. (2018). TGF-β downregulation-induced cancer cell death is finely regulated by the SAPK signaling cascade. Exp. Mol. Med. *50*, 1–19.

34. Gibert, B., Eckel, B., Fasquelle, L., Moulin, M., Bouhallier, F., Gonin, V., Mellier, G., Simon, S., Kretz-Remy, C., Arrigo, A.-P., and Diaz-Latoud, C. (2012). Knock down of heat shock protein 27 (HspB1) induces degradation of several putative client proteins. PLoS One *7*, e29719.

35. Oh, S., Kim, E., Kang, D., Kim, M., Kim, J.H., and Song, J.J. (2013). Transforming growth factor-beta gene silencing using adenovirus expressing TGF-beta1 or TGF-beta2 shRNA. Cancer Gene Ther. *20*, 94–100.

36. Kim, J., Kim, S.Y., Kang, S., Yoon, H.R., Sun, B.K., Kang, D., Kim, J.H., and Song, J.J. (2012). HSP27 modulates survival signaling networks in cells treated with curcumin and TRAIL. Cell. Signal. *24*, 1444–1452.

37. Mahmud, I., and Liao, D. (2019). DAXX in cancer: phenomena, processes, mechanisms and regulation. Nucleic Acids Res. *47*, 7734–7752.

38. Schreiner, S., Bürck, C., Glass, M., Groitl, P., Wimmer, P., Kinkley, S., Mund, A., Everett, R.D., and Dobner, T. (2013). Control of human adenovirus type 5 gene expression by cellular Daxx/ATRX chromatin-associated complexes. Nucleic Acids Res. *41*, 3532–3550.

39. Kang, S., Kang, D., Bakhtiar Ul Islam, S.M., Je, S., Kim, J.H., and Song, J.J. (2015). Silencing Daxx increases the anti-tumor activity of a TRAIL/shRNA Bcl-xL-expressing oncolytic adenovirus through enhanced viral replication and cellular arrest. Cell. Signal. *27*, 1214–1224.

40. Lee, J., Oh, G.H., Hong, J.A., Choi, S., Choi, H.J., and Song, J.J. (2021). Enhanced oncolytic adenoviral production by downregulation of death-domain associated protein and overexpression of precursor terminal protein. Sci. Rep. *11*, 856–878.

41. Parker Kerrigan, B.C., Shimizu, Y., Andreeff, M., and Lang, F.F. (2017). Mesenchymal stromal cells for the delivery of oncolytic viruses in gliomas. Cytotherapy *19*, 445–457.

42. Ferguson, M.S., Lemoine, N.R., and Wang, Y. (2012). Systemic delivery of oncolytic viruses: hopes and hurdles. Adv. Virol. *2012*, 805629. https://doi.org/10.1155/2012/805629.

43. Kang, S., Kim, J.H., Kim, S.Y., Kang, D., Je, S., and Song, J.J. (2014). Establishment of a mouse melanoma model system for the efficient infection and replication of human adenovirus type 5-based oncolytic virus. Biochem. Biophys. Res. Commun. *453*, 480–485.

44. Querido, E., Blanchette, P., Yan, Q., Kamura, T., Morrison, M., Boivin, D., Kaelin, W.G., Conaway, R.C., Conaway, J.W., and Branton, P.E. (2001). Degradation of p53 by adenovirus E4orf6 and E1B55K proteins occurs via a novel mechanism involving a Cullin-containing complex. Genes Dev. *15*, 3104–3117.

45. Cheng, C.Y., Gilson, T., Wimmer, P., Schreiner, S., Ketner, G., Dobner, T., Branton, P.E., and Blanchette, P. (2013). Role of E1B55K in E4orf6/E1B55K E3 ligase complexes formed by different human adenovirus serotypes. J. Virol. *87*, 6232–6245.

46. Royds, J.A., Hibma, M., Dix, B.R., Hananeia, L., Russell, I.A., Wiles, A., Wynford-Thomas, D., and Braithwaite, A.W. (2006). p53 promotes adenoviral replication and increases late viral gene expression. Oncogene *25*, 1509–1520.

47. Cencioni, C., Capogrossi, M.C., and Napolitano, M. (2012). The SDF-1/CXCR4 axis in stem cell preconditioning. Cardiovasc. Res. *94*, 400–407.

48. Jin, W., Liang, X., Brooks, A., Futrega, K., Liu, X., Doran, M.R., Simpson, M.J., Roberts, M.S., and Wang, H. (2018). Modelling of the SDF-1/CXCR4 regulated in vivo homing of therapeutic mesenchymal stem/stromal cells in mice. PeerJ *6*, e6072.

49. Guo, J., Lin, G., Bao, C., Hu, Z., Chu, H., and Hu, M. (2008). Insulin-like growth factor 1 improves the efficacy of mesenchymal stem cells transplantation in a rat model of myocardial infarction. J. Biomed. Sci. *15*, 89–97.

50. Youssef, A., Aboalola, D., and Han, V.K.M. (2017). The roles of insulin-like growth factors in mesenchymal stem cell niche. Stem Cells Int. *2017*, 9453108. https://doi.org/10.1155/2017/9453108.

51. Lee, J.H., Yoon, Y.M., and Lee, S.H. (2017). Hypoxic preconditioning promotes the bioactivities of mesenchymal stem cells via the HIF-1alpha-GRP78-Akt Axis. Int. J. Mol. Sci. *18*, 1320–1333.

52. Fan, G.C. (2012). Role of heat shock proteins in stem cell behavior. Prog. Mol. Biol. Transl. Sci. *111*, 305–322.

53. Su, R., Li, Z., Li, H., Song, H., Bao, C., Wei, J., and Cheng, L. (2010). Grp78 promotes the invasion of hepatocellular carcinoma. BMC Cancer *10*, 20.

54. Conner, C., Lager, T.W., Guldner, I.H., Wu, M.-Z., Hishida, Y., Hishida, T., Ruiz, S., Yamasaki, A.E., Gilson, R.C., Belmonte, J.C.I., et al. (2020). Cell surface GRP78 promotes stemness in normal and neoplastic cells. Sci. Rep. *10*, 3474.

55. Dauer, P., Sharma, N.S., Gupta, V.K., Durden, B., Hadad, R., Banerjee, S., Dudeja, V., Saluja, A., and Banerjee, S. (2019). ER stress sensor, glucose regulatory protein 78 (GRP78) regulates redox status in pancreatic cancer thereby maintaining "stemness. Cell Death Dis. *10*, 132–144.

56. Ho, I.A.W., Chan, K.Y.W., Ng, W.H., Guo, C.M., Hui, K.M., Cheang, P., and Lam, P.Y.P. (2009). Matrix metalloproteinase 1 is necessary for the migration of human bone marrow-derived mesenchymal stem cells toward human glioma. Stem Cells *27*, 1366–1375.

57. Guan, S.P., Lam, A.T.L., Newman, J.P., Chua, K.L.M., Kok, C.Y.L., Chong, S.T., Chua, M.L.K., and Lam, P.Y.P. (2018). Matrix metalloproteinase-1 facilitates MSC migration via cleavage of IGF-2/IGFBP 2 complex. FEBS Open Bio *8*, 15–26.

58. Sohni, A., and Verfaillie, C.M. (2013). Mesenchymal stem cells migration homing and tracking. Stem Cells Int. *2013*, 130763. https://doi.org/10.1155/2013/130763.

59. Bhoopathi, P., Chetty, C., Gogineni, V.R., Gujrati, M., Dinh, D.H., Rao, J.S., and Lakka, S.S. (2011). MMP-2 mediates mesenchymal stem cell tropism toward medulloblastoma tumors. Gene Ther. *18*, 692–701.

60. Li, H., Song, H., Luo, J., Liang, J., Zhao, S., and Su, R. (2012). Knockdown of glucose-regulated protein 78 decreases the invasion, metalloproteinase expression and ECM degradation in hepatocellular carcinoma cells. J. Exp. Clin. Cancer Res. *31*, 39.

61. Li, Z., and Li, Z. (2012). Glucose regulated protein 78: a critical link between tumor microenvironment and cancer hallmarks. Biochim. Biophys. Acta *1826*, 13–22.

62. Russell, S.J., and Barber, G.N. (2018). Oncolytic viruses as antigen-agnostic cancer vaccines. Cancer Cell *33*, 599–605.

63. Atasheva, S., Emerson, C.C., Yao, J., Young, C., Stewart, P.L., and Shayakhmetov, D.M. (2020). Systemic cancer therapy with engineered adenovirus that evades innate immunity. Sci. Transl. Med. *12*, eabc6659.

64. Zhang, J., Chen, H., Chen, C., Liu, H., He, Y., Zhao, J., Yang, P., Mao, Q., and Xia, H. (2021). Systemic administration of mesenchymal stem cells loaded with a novel oncolytic adenovirus carrying IL-24/endostatin enhances glioma therapy. Cancer Lett. *509*, 26–38.

65. Yoon, A.-R., Hong, J., Li, Y., Shin, H.C., Lee, H., Kim, H.S., and Yun, C.-O. (2019). Mesenchymal stem cell–mediated delivery of an oncolytic adenovirus enhances antitumor efficacy in hepatocellular carcinoma. Cancer Res. *79*, 4503–4514.

66. Fischer, U.M., Harting, M.T., Jimenez, F., Monzon-Posadas, W.O., Xue, H., Savitz, S.I., Laine, G.A., and Cox, C.S., Jr. (2009). Pulmonary passage is a major obstacle for intravenous stem cell delivery: the pulmonary first-pass effect. Stem Cells Dev. *18*, 683–692.

67. Krueger, T.E.G., Thorek, D.L.J., Denmeade, S.R., Isaacs, J.T., and Brennen, W.N. (2018). Concise review: mesenchymal stem cell-based Drug delivery: the good, the bad, the ugly, and the promise. Stem Cells Transl. Med. *7*, 651–663.

68. Prestwich, R.J., Errington, F., Diaz, R.M., Pandha, H.S., Harrington, K.J., Melcher, A.A., and Vile, R.G. (2009). The case of oncolytic viruses versus the immune system: waiting on the judgment of Solomon. Hum. Gene Ther. *20*, 1119–1132.

69. Blair, G.E., Dixon, S.C., Griffiths, S.A., and Zajdel, M.E. (1989). Restricted replication of human adenovirus type 5 in mouse cell lines. Virus Res. *14*, 339–346.

70. Ying, B., Toth, K., Spencer, J.F., Meyer, J., Tollefson, A.E., Patra, D., Dhar, D., Shashkova, E.V., Kuppuswamy, M., Doronin, K., et al. (2009). INGN 007, an oncolytic adenovirus vector, replicates in Syrian hamsters but not mice: comparison of biodistribution studies. Cancer Gene Ther. *16*, 625–637.

OMTO, Volume *27*

Supplemental information

Enhanced tumor targeting and timely viral release of mesenchymal stem cells/oncolytic virus complex due to GRP78 and inducible E1B55K expressions greatly increase the antitumor effect of systemic treatment

Soojin Choi, Jeong A. Hong, Hye Jin Choi, and Jae J. Song

Table S1 List of PCR primers for subcloning

Vector Name	Forward sequence	Reverse sequence
pVAX1-3484-RFP	CCGGGTCGACGACATTGATT	GATTGTCGACCCATAGAGCC
pcDNA3.1-Hygro-E1B55K	GCGGATCCATGGAGCGAAGAAACCCATCT	GAGCGGCCGCTCAATCTGTATCTTCATCGCT
pFlag-CMV2-E4orf6	GTACAAGCTTATGACTACGTCCGGCGTTCC	CACCTCTAGACTACATGGGGGTAGAGTCAT
pCA14-E1B55K	TTCATCTAGAATGGAGCGAAGAAACCCATC	GACGAAGCTTTCAATCTGTATCTTCATCGC
pcDNA3.1-Hygro-GRP78	GATTCTCGAGATGAAGCTCTCCCTGG	GGCCTCTAGACTACAACTCATCTTTT
pRetro-Tetone-Puro-E1B55K	CCTCGTAAAGAATTCATGGAGCGAAGAAACC CATCTGAG	GAGGTGGTCTGGATCCTCAATCTGTATCTTCAT CGCTAGA

Table S2 List of qRT-PCR primers

Primer name	Forward sequence	Reverse sequence
Vimentin	TGGATTCACTCCCTCTGGTT	GGTCATCGTGATGCTGAGAA
β-catenin	TCTGAGGACAAGCCACAAGATTACA	TGGGCACCAATATCAAGTCCAA
HDAC6	AAGAAGACCTAATCGTGGGACT	GCTGTGAACCAACATCAGCTC
Daxx	GACGGACATTTCCTCTTCCA	TCTCATGCACTGACCTTTGC
Akt1	TCCTCCTCAAGAATGATGGCA	GTGCGTTCGATGACAGTGGT
MAPKAPK2	CAGCAGTTCCCGCAGTTC	CGAATTTCTCCTGGGTCCTC
PTEN	ACCAGAGACAAAAAGGGAGTA	ACCACAAACTGAGGATTGCA
TRAF6	CCTTTGGCAAATGTCATCTGTG	CTCTGCATCTTTTCATGGCAAC
STAT3	TATGCGGCCAGCAAAGAATCA	CGGGCAATCTCCATTGGCT
HSF1	GCCTTCCTGACCAAGCTGT	AAGTACTTGGGCAGCACCTC
HSP27	CTACATCTCCCGGTGCTTCA	GGATGGTGATCTCGTTGGAC
TGF-β1	CAAGGGCTACCATGCCAACT	AGGGCCAGGACCTTGCTG
GRP78	CTTGCCGTTCAAGGTGGTTG	GCATGGGTAACCTTCTTTCC
β-actin	ACTCTTCCAGCCTTCCTTC	ATCTCCTTCTGCATCCTGTC
E4orfB region	CGTGGTCAAACTCTACAGCC	GCATGAGCATGACTACGATG

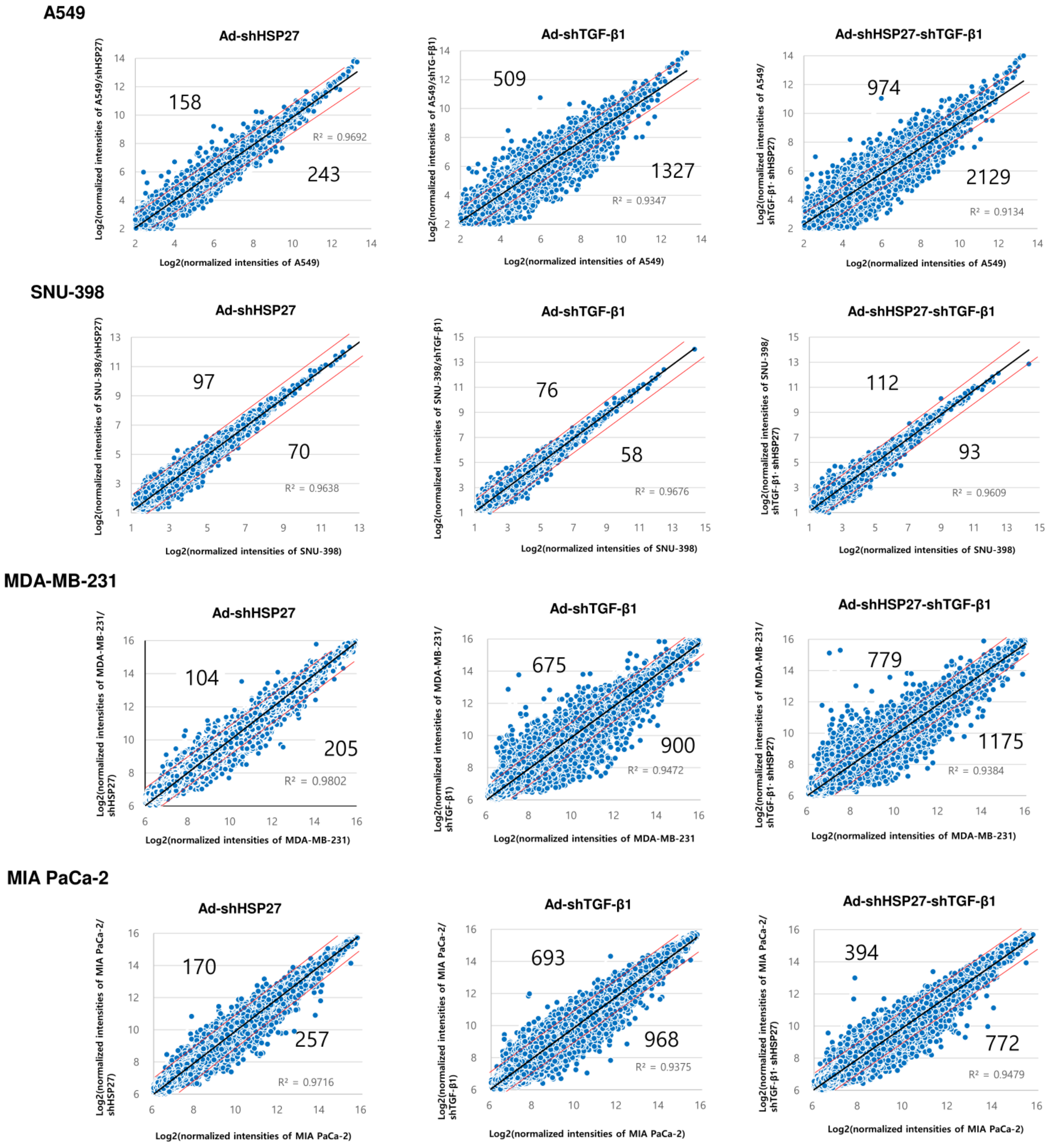

Figure S1 Scatter plots of relative probe set intensities after downregulation of HSP27, TGF-β1 or HSP27-TGF-β1 compared to before downregulation of HSP27, TGF-β1 or HSP27-TGF-β1, respectively, from four different cancer cells (A549, SNU-398, MDA-MB-231, MIA PaCa-2)
Normalized probe set intensities of before or after downregulation of HSP27, TGF-β1 or HSP27-TGF-β1 are plotted. The numbers indicate over 2-fold variation in intensities outside of two diagonal lines (red).

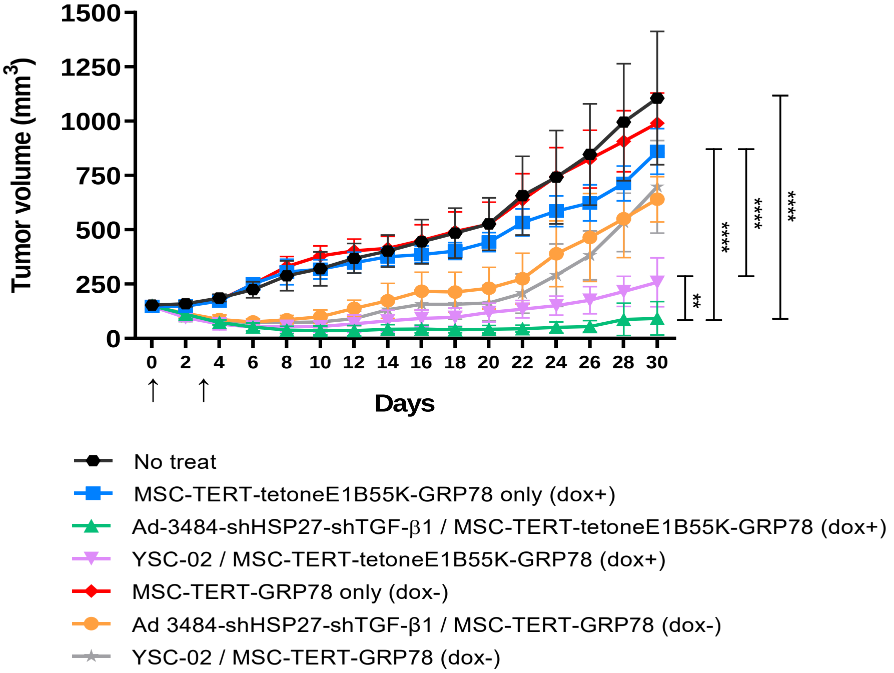

Figure S2 Tumor volume measurement in A549 xenograft models
8×10^6 A549 cells were implanted subcutaneously in the flank region of BALB/c athymic nude mice, and either MSC-TERT-GRP78 or MSC-TERT-tetoneE1B55K-GRP78 loaded with oncolytic adenovirus was injected intravenously twice with an interval of 3 days. Tumor volume was monitored and recorded every 2 days until the end of the study. Arrows indicate when the MSC or oncolytic adenovirus/MSC complex was administered. Error bars represent mean ± SD from 10 mice per group: **P<0.01, ****P<0.0001.
